# Discovery and mechanism of K63-linkage-directed deubiquitinase activity in USP53

**DOI:** 10.1038/s41589-024-01777-0

**Published:** 2024-11-25

**Authors:** Kim Wendrich, Kai Gallant, Sarah Recknagel, Stavroula Petroulia, Nafizul Haque Kazi, Jan André Hane, Siska Führer, Karel Bezstarosti, Rachel O’Dea, Jeroen Demmers, Malte Gersch

**Affiliations:** 1 Chemical Genomics Centre, Max Planck Institute of Molecular Physiology, Dortmund, Germany; 2 Department of Chemistry and Chemical Biology, TU Dortmund University, Dortmund, Germany; 3 Proteomics Center, Erasmus University Medical Center, Rotterdam, The Netherlands; 4 Present Address: Medical Research Council Protein Phosphorylation and Ubiquitylation Unit, University of Dundee, Dundee, UK

**Keywords:** Post-translational modifications, Post-translational modifications, X-ray crystallography, Proteomics, Proteases

## Abstract

Ubiquitin-specific proteases (USPs) represent the largest class of human deubiquitinases (DUBs) and comprise its phylogenetically most distant members USP53 and USP54, which are annotated as catalytically inactive pseudoenzymes. Conspicuously, mutations within the USP domain of *USP53* cause progressive familial intrahepatic cholestasis. Here, we report the discovery that USP53 and USP54 are active DUBs with high specificity for K63-linked polyubiquitin. We demonstrate how *USP53* mutations abrogate catalytic activity, implicating loss of DUB activity in *USP53*-mediated pathology. Depletion of *USP53* increases K63-linked ubiquitination of tricellular junction components. Assays with substrate-bound polyubiquitin reveal that USP54 cleaves within K63-linked chains, whereas USP53 can en bloc deubiquitinate substrate proteins in a K63-linkage-dependent manner. Biochemical and structural analyses uncover underlying K63-specific S2 ubiquitin-binding sites within their catalytic domains. Collectively, our work revises the annotation of USP53 and USP54, provides reagents and a mechanistic framework to investigate K63-linked polyubiquitin decoding and establishes K63-linkage-directed deubiquitination as a new DUB activity.

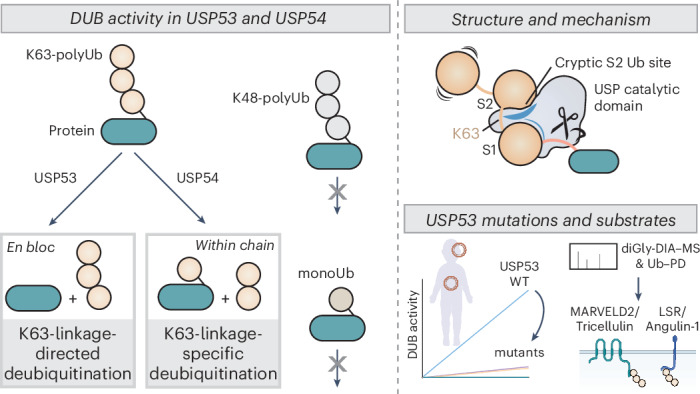

## Abstract (web summary)

Wendrich, Gallant and colleagues find that USP53 and USP54 are active deubiquitinases, with USP53 removing ubiquitin chains from substrate proteins in a chain-linkage-directed manner, and provide biochemical and structural insights into their mechanism, cellular substrates and disease implications.

## Main

The covalent attachment of the small protein ubiquitin to substrates and to other ubiquitin moieties facilitates the formation of a diverse array of post-translational modifications; this attachment is characterized by structural and functional heterogeneity and collectively termed the ‘ubiquitin code’ (refs. ^1,2^). In addition to monoubiquitination, polyubiquitin chains of eight linkages exist, with K48-linked and K63-linked chains being the most abundant in human cells^1,3^. While the attachment of K48-linked ubiquitin chains to substrates commonly facilitates proteasomal degradation, predominantly nondegradative roles have been described for K63-linked chains, including in intracellular trafficking, innate immune signaling and genome maintenance^4–6^. Ubiquitin chain length encodes an additional layer of information^1,2,7–13^. However, in contrast to K11-linked and K48-linked polyubiquitin chains^9,14–17^, molecular mechanisms for length-dependent decoding of K63-linked chains have remained elusive.

Deubiquitinating enzymes (DUBs) edit ubiquitin chains or cleave ubiquitin–substrate linkages, thereby regulating the ubiquitination status of proteins to determine their stability, localization, activity and function^1,18–21^. The human genome encodes approximately 100 DUBs, which can be classified into ubiquitin linkage-specific and nonspecific enzymes^11,15,22–24^. High ubiquitin linkage specificity has been demonstrated for members of the ovarian tumor protease (OTU; diverse linkages)^15^, MINDY (K48)^22^, ZUFSP (K63)^6,11,23,25^ and JAMM (K63)^26–28^ families. In contrast, DUBs of the ubiquitin C-terminal hydrolase (UCH), Josephin and ubiquitin-specific protease (USP) families generally show poor discrimination of linkages^24^. Of those, USPs form the largest and most diverse DUB family with ~60 members in humans^29^.

USP53 and USP54 are poorly characterized USP family members^30,31^. They possess high homology within their predicted catalytic domains but show otherwise very low sequence homology to all other USP members^20^. Notably, both proteins have been reported to be catalytically inactive^20,32^ because of the absence of strongly conserved residues before their catalytic histidines^32^, the inability of bacterially expressed USP53 to hydrolyze a monoubiquitin substrate^33^ and the lack of reactivity of cellular USP53 with a ubiquitin probe^30^. However, we were intrigued by recent reports that identified loss or biallelic mutations in *USP53* as the cause for progressive familial intrahepatic cholestasis, a hereditary liver disorder in children^34–37^. This phenotype and proposed cellular roles linked to USP53 and USP54 have not been reconciled with their annotation as pseudoenzymes^31^. In addition, the lack of an in vitro assessment of catalytic activity and unavailable structural information have complicated insights into pathology-related molecular mechanisms.

Here, we report USP53 and USP54 to be active DUBs with specific cleavage activities on K63-linked ubiquitin chains, revising their annotation as inactive USP family members. Biochemical experiments and a crystal structure of USP54 in complex with a K63-linked diubiquitin probe uncover cryptic S2 ubiquitin sites within the USP domains of both enzymes, underlying efficient cleavage within longer K63-linked chains by USP54. USP53 catalyzes K63-linkage-directed en bloc deubiquitination, a DUB activity previously not observed, which is driven by a K63-specific S2 site within its catalytic domain. Taken together, our data establish distinct molecular mechanisms of length-dependent decoding of K63-linked polyubiquitin chains and mechanistically connect the loss of USP53 activity to pediatric cholestasis.

## Results

### USP53 and USP54 are active DUBs with K63-linked polyubiquitin specificity

**Fig. 1:**
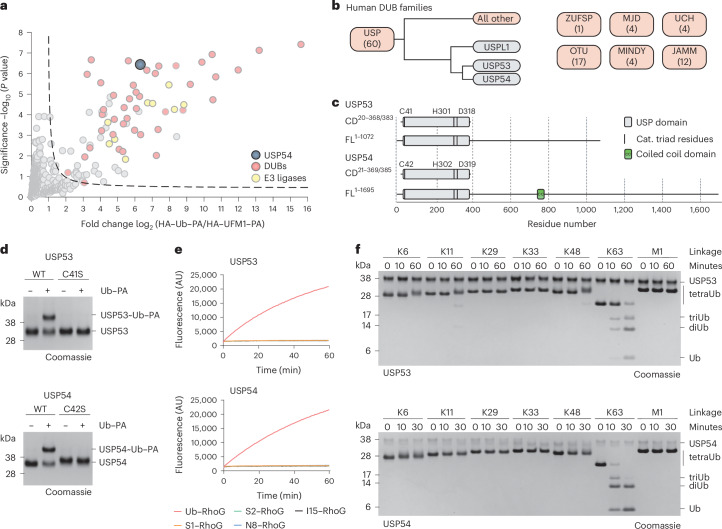
USP53 and USP54 are active DUBs for K63-linked polyubiquitin. **a**, Proteomics analysis of ubiquitin–PA probe-labeled proteins in HeLa cell lysate. The volcano plot shows the enrichment (fold change) of proteins detected in HA pulldowns from lysate treated with HA–ubiquitin–PA probe in comparison to HA–UFM1–PA as a control. Identified proteins are shown as dots, HECT E3 ligases are shown in yellow, DUBs are shown in red and USP54 is shown in blue. **b**, Human DUB families are shown as boxes with numbers of members given in brackets. USP53 and USP54 are annotated as inactive and comprise the most distantly related catalytic domains within the USP family, with highest similarity to the deSUMOylase USPL1. **c**, Domain architectures of human full-length (FL) and catalytic domain (CD) constructs of USP53 and USP54 used in this study. **d**, Probe reactivity assay with recombinant catalytic domains. HA–ubiquitin–PA was incubated with wild-type (WT) USP53^20–383^ and USP54^21–369^ or inactive mutants of USP53^20–383^ and USP54^21–385^. Probe reactivity was analyzed by SDS–PAGE and Coomassie staining. **e**, Ubiquitin and Ubl RhoG cleavage assay. USP53^20^^–^^383^ (200 nM, top) or USP54^21^^–^^369^ (200 nM, bottom) was added to ubiquitin–RhoG (Ub–RhoG), SUMO1–RhoG (S1–RhoG), SUMO2–RhoG (S2–RhoG), NEDD8–RhoG or ISG15–RhoG (I15–RhoG) and the fluorescence was recorded. Data are shown as the average of technical triplicates. **f**, Gel-based ubiquitin chain cleavage assay. Specifically linked tetraubiquitin chains (2 µM) were incubated with USP53^20^^–^^383^ (3 µM) or USP54^21^^–^^369^ (300 nM). Samples were taken after the indicated time points and cleavage activity was analyzed by SDS–PAGE and Coomassie staining. diUb, diubiquitin; triUb, triubiquitin; tetraUb, tetraubiquitin. Source data

**Extended Data Fig. 1:**
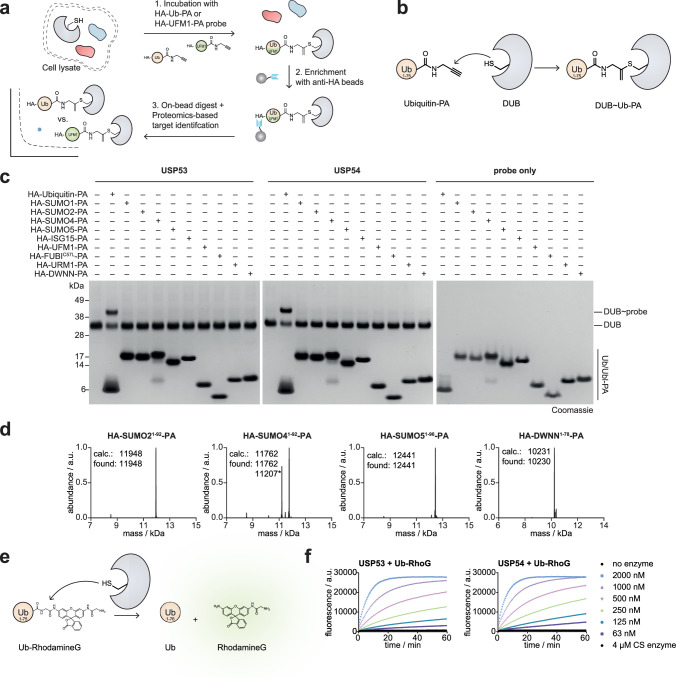
USP53 and USP54 are active DUBs. **a**. Schematic of the workflow used to identify active DUBs. HeLa cell lysate was treated with HA-Ub-PA or HA-UFM1-PA probe. Labelled proteins were enriched and detected by mass spectrometry. **b**. Schematic of the probe reactivity assay. The C-terminal propargylamine (PA) warhead reacts with the catalytic cysteine of DUBs to a vinyl thioether, forming a covalent DUB~Ub-PA complex. **c**. Ub/Ubl specificity assayed through probe reactivity with recombinant catalytic domains. A panel of HA-Ub/Ubl-PA probes was incubated with wild-type USP53^20-383^ or wild-type USP54^21-369^. Probe reactivity was analyzed by SDS-PAGE and Coomassie staining. Uncropped versions of all gels are provided as Source Data. **d**. Intact protein mass spectrometry of probes used in panel c. Expected and found masses are given in Dalton. The star denotes an HA-SUMO4^1–92^ species N-terminally truncated by four residues. All other probes have been characterized previously^41^. **e**. Schematic of the Ubiquitin-RhodamineG (Ub-RhoG) cleavage assay. DUBs cleave the amide bond between ubiquitin and RhoG and thereby liberate fluorescent RhoG. **f**. Ub-RhoG cleavage assay. Indicated concentrations of USP53^20-383^ (*left*) and USP54^21-369^ (*right*) or of the catalytic inactive mutants were added to Ub-RhoG and fluorescence was recorded. Data are shown as average of technical triplicates. CS enzymes denote the use of catalytic cysteine to serine mutated enzymes as in Fig. 1d. Of note, this activity is approximately 3 to 4 orders of magnitude weaker than for other USP DUBs including USP2, USP7, USP36 and USP42^41^. Source data

Activity-based probes are commonly used for the identification, activity profiling and structural analysis of DUBs^11,22,23,38^. We recently used propargylamide (PA)-based probes^39,40^, whose C-terminal warhead can form a vinyl thioether with the catalytic cysteines of active enzymes, to characterize ubiquitin and ubiquitin-like protein (Ubl) cross-reactivity of DUBs (Extended Data Fig. 1a,b)^41^. Consistent with previous studies, the ubiquitin probe facilitated the enrichment of many DUBs and HECT family E3 ligases^38^. Surprisingly, we also detected prominent enrichment of USP54 (Fig. 1a) even though this enzyme and its close homolog USP53 are widely reported to be catalytically inactive^20,30,33^. On the basis of the catalytic domains, both enzymes are the phylogenetically most distinct members of the USP family of DUBs and cluster with the deSUMOylase USPL1 (Fig. 1b)^20^.

To test for the potential activity of USP53 and USP54, we bacterially expressed and purified their catalytic domains (Fig. 1c). Using a panel of probes including ubiquitin, different SUMO orthologs and other Ubls (Extended Data Fig. 1c,d), we observed specific reactivity with HA–ubiquitin–PA for both USP53 and USP54 (Extended Data Fig. 1c). This reactivity depended on the presence of the predicted catalytic cysteines (Fig. 1d). To directly test for ubiquitin C-terminal hydrolase activity, we used a panel of fluorogenic reagents derived from ubiquitin and Ubls (Extended Data Fig. 1e). We observed concentration-dependent and specific cleavage of ubiquitin–RhoG (rhodamine G) but not of SUMO1, SUMO2, ISG15 or NEDD8 reagents by both USP53 and USP54 (Fig. 1e and Extended Data Fig. 1f).

With the notable exception of CYLD, USP DUBs generally show unspecific cleavage or only moderate selectivity toward differently linked ubiquitin chains^24,42,43^. To analyze cleavage activity on isopeptide linkages, we assayed a tetraubiquitin panel. To our surprise, USP53 and USP54 cleaved K63-linked chains with remarkable specificity (Fig. 1f). This finding was highly unexpected as no other human USP DUB shows specific cleavage activity of a single linkage. For USP54, no cleavage was observed for any other linkage, whereas USP53 minimally cleaved K11-linked and K48-linked tetraubiquitin chains at longer time points. Collectively, these experiments demonstrate that human USP53 and USP54 are active enzymes with a specificity for K63-linked polyubiquitin (Fig. 1 and Extended Data Fig. 1).

### Mutations in *USP53* cause loss of catalytic activity

**Fig. 2:**
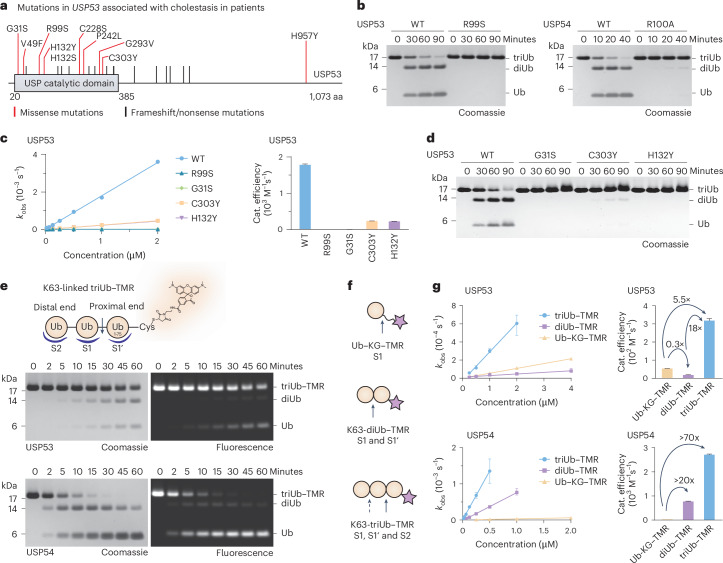
Analysis of *USP53* mutations and polyubiquitin chain-length-dependent cleavage. **a**, Schematic of human USP53 with amino acid substitutions associated with cholestasis or hearing loss. Single amino acid changes cluster in the catalytic domain. **b**, Gel-based ubiquitin chain cleavage assay. K63-linked triubiquitin (3 µM) was incubated with USP53^20–383^ (2 µM, left) or USP54^21^^–^^369^ (300 nM, right). Cleavage was analyzed by SDS–PAGE and Coomassie staining. **c**, Ubiquitin–RhoG cleavage assay. Ubiquitin–RhoG was incubated with USP53^20^^–^^383^ (WT or indicated mutants; raw data in Extended Data Fig. 2a) and the fluorescence was recorded. Observed rate constants (*k*_obs_, shown as the mean ± s.e.m.) were plotted over enzyme concentrations to obtain catalytic efficiencies (shown as the mean ± s.e.m.). **d**, Gel-based ubiquitin chain cleavage assay with additional *USP53* mutations, shown as in **b**. **e**, Fluorescence-based triubiquitin cleavage assay. A K63-linked triubiquitin substrate (3 µM) was used, in which the proximal Ub^1^^–^^75^-CA was conjugated to maleimide–TAMRA (triubiquitin–TMR). Cleavage by USP53^20^^–^^383^ (3 µM) or USP54^21^^–^^369^ (300 nM) was analyzed by SDS–PAGE, in-gel fluorescence and Coomassie staining. The major cleavage position is indicated with an arrow. S2, S1 and S1′ ubiquitin-binding sites in the DUB were assigned consistent with the observed cleavage products. **f**, Schematic of ubiquitin substrates used in **g**. Cleavage sites are indicated by arrows. Ubiquitin-binding sites, which lead to cleavage when engaged, are given for the respective substrates. TAMRA is shown as a purple star. Structures of the substrates are shown in Extended Data Fig. 3. **g**, Fluorescence polarization cleavage assays. Substrates were incubated with USP53^20^^–^^383^ (top) or USP54^21^^–^^369^ (bottom) and fluorescence anisotropy was recorded (raw data in Extended Data Fig. 3i). Observed rate constants (*k*_obs_, shown as the mean ± s.e.m.) were plotted over enzyme concentrations to obtain catalytic efficiencies (shown as the mean ± s.e.m.). Source data

**Extended Data Fig. 2:**
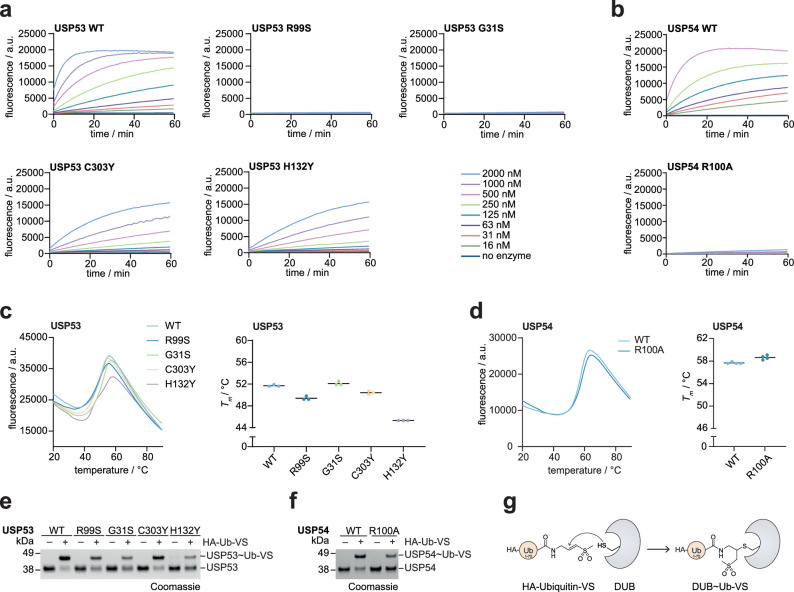
Cholestasis-associated patient mutations of USP53 are catalytically impaired. **a**. Ub-RhoG cleavage assay. Indicated concentrations of USP53^20-383^ (WT or patient mutants) were added to Ub-RhoG and fluorescence was recorded over time. Data are shown as average of technical triplicates. **b**. Ub-RhoG cleavage assay with USP54^21-385^, shown as in panel a. **c-d**. Protein stability assessment of USP53^20-383^ (panel c) or USP54^21-369^ (panel d) proteins by thermal shift analysis. Averaged fluorescence raw data are shown and melting temperatures (*T*_*m*_) are plotted from technical triplicates (USP53) or quadruplicates (USP54). **e-f**. Probe reactivity assay. HA-Ub-VS was incubated with indicated USP53^20-383^ (panel e) or USP54^21-369^ (panel f) proteins. Probe reactivity was analyzed by SDS-PAGE and Coomassie staining. Uncropped versions of all gels are provided as Source Data. **g**. Schematic of the HA-Ub-VS probe reactivity assay. Source data

Recently, 14 reports have linked pediatric cholestasis to biallelic mutations in the *USP53* gene (Supplementary Table 1)^34–37^. However, the falsely presumed enzymatic inactivity of the USP53 protein has so far prevented a mechanistic analysis of the underlying pathologies. We compiled all missense mutations in *USP53* and found a clustering within its catalytic domain (Fig. 2a). We obtained the USP53 catalytic domain with the disease-associated substitution R99S through bacterial expression and found it to be completely inactive toward K63-linked triubiquitin chains (Fig. 2b) and ubiquitin–RhoG (Fig. 2c and Extended Data Fig. 2a). Consistently, USP54 with an equivalent substitution of R100A was virtually inactive in the same assays (Fig. 2b and Extended Data Fig. 2b). In addition, we tested the G31S, C303Y and H132Y USP53 substitutions in the same assays (Fig. 2c,d and Extended Data Fig. 2) and found them to be strongly impaired in catalysis, particularly in ubiquitin chain cleavage (Fig. 2d). All mutant proteins were folded as assessed in thermal shift assays (TSAs) (Extended Data Fig. 2c,d) and were capable of recognizing ubiquitin, as evident from reduced but retained reactivity toward a ubiquitin probe (Extended Data Fig. 2e–g). These data demonstrate that disease-associated mutations abrogate the enzymatic activity of USP53. More broadly, the data implicate the loss of USP53 DUB activity as the cause of *USP53*-mediated pathology.

### Ubiquitin chain length decoding by USP53 and USP54

**Extended Data Fig. 3:**
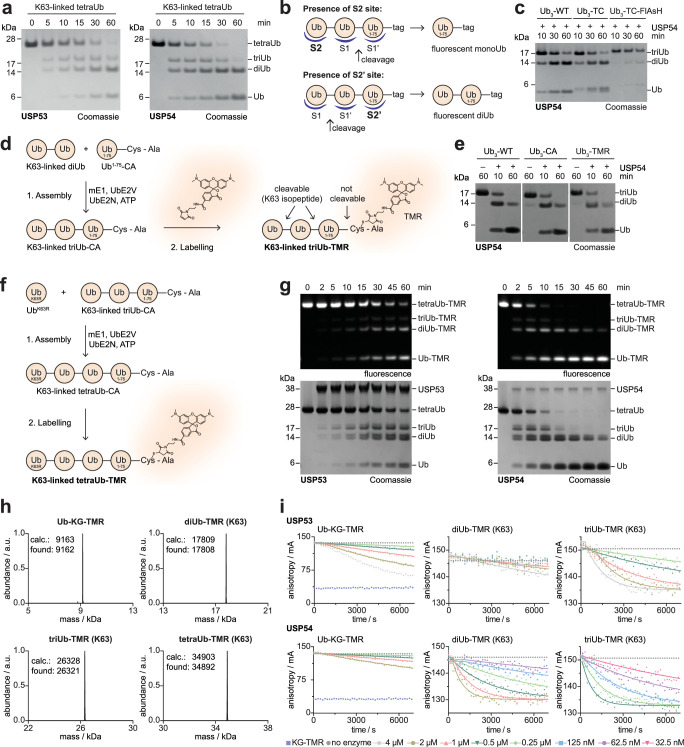
An S2 site, but not an S3 site, underlies USP53 and USP54 polyubiquitin cleavage. **a**. Time-resolved gel-based cleavage assays of K63-linked tetraubiquitin (2 µM) by USP53^20-383^ (4 µM, *left*) and USP54^21-369^ (300 nM, *right*). Cleavage activity was analyzed by SDS-PAGE and Coomassie staining. Uncropped versions of all gels are provided as Source Data. **b**. Schematic of expected products of a triubiquitin cleavage assay in which the reagent contains a non-cleavable, C-terminal fluorescent label. Ubiquitin binding sites in a DUB are shown, expected cleavage sites are indicated with arrows. The presence of an S2 site would lead to fluorescently labeled monoubiquitin, whereas the presence of an S2’ site would lead to fluorescently labeled diubiquitin. **c**. Gel-based cleavage assay with a FlAsH-based reagent. Native K63-linked triubiquitin chains, K63-linked triubiquitin with a C-terminal tetracysteine (TC) tag in the proximal Ubiquitin (Ub_3_-TC) and FlAsH-labeled triubiquitin (Ub_3_-TC-FlAsH, all at 3 µM) were incubated with USP54^21-369^ (300 nM) for indicated times. USP54 activity was impaired by the presence of the FlAsH dye. **d**. Schematic of the generation of the TAMRA-based, fluorescent K63-linked triubiquitin substrate. TMR, TAMRA. **e**. Gel-based cleavage assay with a TAMRA-based reagent. Equivalent assays as shown in panel c with K63-linked triubiquitin chains shown in panel d (3 µM substrate, 300 nM enzyme). **f**. Schematic of the generation of the TAMRA-based, fluorescent K63-linked tetraubiquitin substrate. **g**. Gel-based cleavage assay. Fluorescent K63-linked tetraubiquitin (3 µM) was incubated with USP53^20-383^ (4 µM) or USP54^21-369^ (300 nM). Cleavage activity was analyzed as in Fig. 2e. The equally formed fluorescent diubiquitin and monoubiquitin species demonstrate that neither USP53 nor USP54 possess S3 ubiquitin binding sites. **h**. Deconvoluted intact protein mass spectra of indicated reagents. Calculated and observed molecular weights are given in Dalton. **i**. Fluorescence polarization cleavage assays of USP53^20-383^ and USP54^21-369^ and indicated reagents. Catalytic efficiencies derived from these data are shown in Fig. 2g. Source data

Strikingly, diubiquitin chains accumulated during K63-linked polyubiquitin chain cleavage by both enzymes (Figs. 1f and 2b), as confirmed in further time-resolved assays (Extended Data Fig. 3a). These data suggest an unusual ubiquitin chain length preference of both enzymes. The linkage specificity of DUBs is typically facilitated by S1 and S1′ ubiquitin-binding sites, with the preferred cleavage of longer chains being facilitated through additional ubiquitin-binding sites (Fig. 2e)^15,22^. To determine whether USP53 and USP54 contain S2′ or S2 sites, we prepared fluorescently labeled K63-linked triubiquitin substrates allowing the separate detection of fluorescent species (Extended Data Fig. 3b–e). As we found USP54 activity to be impaired by the presence of the FlAsH dye (Extended Data Fig. 3c), we generated K63-linked triubiquitin–TAMRA in which the proximal ubiquitin is fluorescently labeled through a C-terminal cysteine (Extended Data Fig. 3d,e). Assaying the cleavage of the reagent by USP53 and USP54 at early time points revealed preferential conversion into nonfluorescent diubiquitin and fluorescent ubiquitin–TAMRA (Fig. 2e). This cleavage pattern suggested the existence of an S2 ubiquitin-binding site in both enzymes, as depicted in Fig. 2e and Extended Data Fig. 3b. A potential fourth ubiquitin-binding site was excluded upon assaying an equivalent K63-linked tetraubiquitin–TAMRA reagent (Extended Data Fig. 3f,g).

We next aimed to quantify the contributions of the S1, S1′ and S2 ubiquitin-binding sites to USP53 and USP54 activity by using monoubiquitin–KG^TAMRA^ together with K63-linked diubiquitin and triubiquitin reagents in fluorescence polarization assays (Fig. 2f and Extended Data Fig. 3h). Cleavage activity was measured as the decline in anisotropy over time (Extended Data Fig. 3i) and was quantified through observed rate constants, yielding catalytic efficiencies of USP53 and USP54 for each substrate (Fig. 2g). USP53 showed poor cleavage of ubiquitin–KG^TAMRA^ and an even lower turnover of the diubiquitin substrate. Addition of a third ubiquitin led to an 18-fold increase in catalytic efficiency (Fig. 2g). This behavior could be explained by competing and catalytically unproductive binding of the monoubiquitin and diubiquitin substrates to a high-affinity S2 binding site. In contrast, USP54 showed an even poorer cleavage of ubiquitin–KG^TAMRA^ but a more than 20-fold increase in catalytic efficiency for diubiquitin–TAMRA. Additional engagement of the S2 site by triubiquitin–TAMRA led to a further increase in catalytic efficiency (Fig. 2g), consistent with the results of the tetraubiquitin panel (Fig. 1f).

### USP53 displays linkage-directed deubiquitination activity

**Fig. 3:**
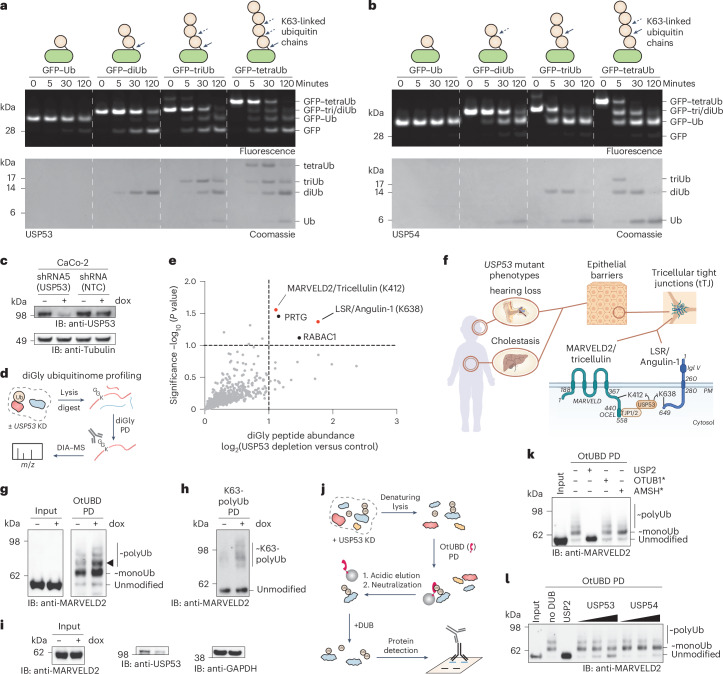
USP53 shows K63-linkage-directed deubiquitination activity. **a**,**b**, Cleavage assay with isopeptide-linked, K63-linked monoubiquitinated or polyubiquitinated substrates. GFP–ubiquitin substrates (1 µM) were incubated with USP53^20–383^ (500 nM; **a**) or USP54^21–369^ (300 nM; **b**). In-gel fluorescence was used to visualize GFP species, while Coomassie staining of the same gel was used to visualize ubiquitin chains. Arrows indicate cleavage sites. **c**, USP53 depletion analysis. CaCo-2 cells stably transduced as indicated were analyzed by western blotting after treatment with doxycycline for 72 h. **d**, Schematic illustration of the diglycine ubiquitinome profiling to identify USP53 substrates. **e**, Volcano plot showing log_2_ fold changes of diglycine (ubiquitinated) peptides upon depletion of USP53 in CaCo-2 cells. Proteins linked to phenotypes similar to *USP53* mutations are indicated in red. Diglycine sites were unambiguously identified. **f**, Model linking *USP53*-associated phenotypes to USP53-modulated ubiquitination of tricellular tight junction proteins (with domains, ubiquitination sites and residue numbers given). PM, plasma membrane. **g**, MARVELD2 ubiquitination analysis through OtUBD pulldown. CaCo-2 shRNA5 (USP53) cells were treated as in **c**, lysates were enriched with the high-affinity ubiquitin binder OtUBD and samples were analyzed by western blotting. The triangle highlights MARVELD2 modified with two ubiquitin moieties, emerging upon USP53 depletion. **h**, MARVELD2 polyubiquitination analysis with K63-specific tUIM^Rap80^ (Rx3A7) TUBE pulldown. **i**, Analysis of protein levels for samples processed in **h**. **j**. Illustration of the workflow used to generate eluates of OtUBD pulldowns for UbiCRest assays. Acidic conditions allowed the elution of ubiquitinated proteins, with the biotinylated OtUBD reagent being retained on streptavidin beads. **k**, UbiCRest assay. OtUBD eluates prepared as described in **j** were treated with the indicated DUBs for 1 h at 37 °C and analyzed by western blot. **l**, UbiCRest assay as in **k** with USP53^20–383^ (0.5, 2 and 5 µM) and USP54^21–369^ (0.3, 1 and 3 µM), showing en bloc deubiquitination of cellular MARVELD2 by recombinant USP53. The results after 2-h incubation are shown in Extended Data Fig. 4k. Source data

While DUB activity is typically assayed with free chains, the majority of cellular polyubiquitin is conjugated to protein substrates^1,2^. To test for distinct activity profiles of USP53 and USP54 in the context of protein-bound ubiquitin chains, we generated a panel of ubiquitinated model substrates including isopeptide-linked monoubiquitinated GFP and GFP conjugated to K48-linked or K63-linked chains of different length (Fig. 3a). The isopeptide linkage of ubiquitin to lysine acylation using conjugating enzymes (LACE)-tagged GFP was generated with recently reported methodology^44^ and chains were subsequently assembled. Consistent with the fluorescence polarization assays, USP54 did not turn over GFP–ubiquitin but shortened K63-linked ubiquitin chains to leave a single ubiquitin on GFP (Fig. 3b). This finding highlights the importance of its S1′ ubiquitin-binding site. Substrate turnover increased with chain length, consistent with the identified S2 site.

**Extended Data Fig. 4:**
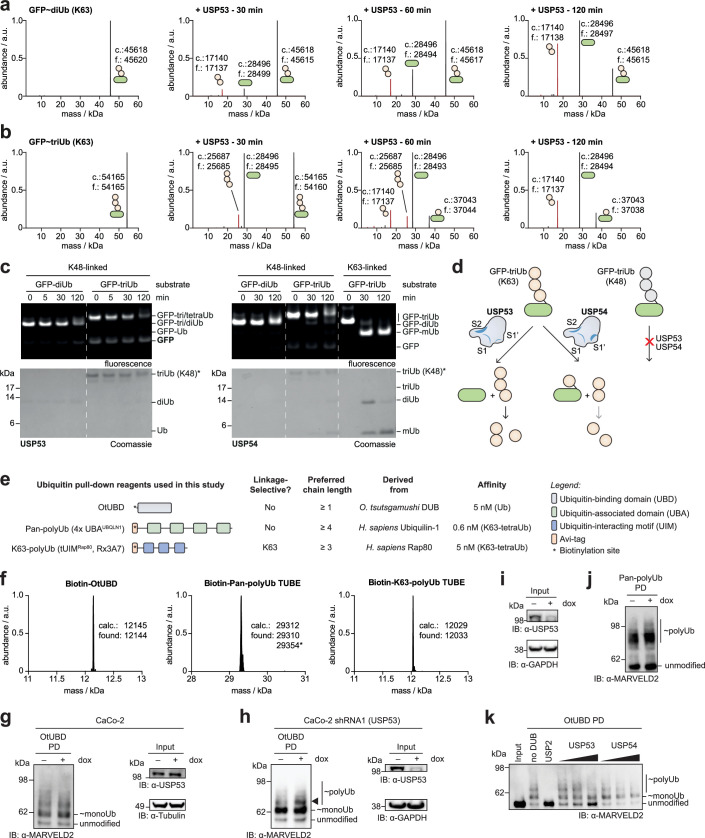
Validation and specificity analysis of linkage-directed deubiquitination activity in USP53. **a**. Intact protein mass spectrometry analysis of GFP-diUb (K63) cleavage, corresponding to Fig. 3a (500 nM USP53, 1 µM substrate). Diubiquitin removed en bloc by USP53 was identified unequivocally. Calculated (c.) and found (f.) protein masses are given in Dalton. **b**. Cleavage analysis of GFP-triUb (K63) by USP53, shown as in panel a. Triubiquitin removed en bloc during the earlier time points was cleaved further to diubiquitin. En bloc removal was observed in the earliest time point. Cleavage within the chain leading to accumulation of a minor amount of monoubiquitinated substrate was observed at later time points, consistent with Fig. 3a. **c**. Cleavage assay for ubiquitinated model substrates modified through isopeptide bonds with K48-linked di- or triubiquitin or with K63-linked triubiquitin. GFP-Ub_n_ substrates (1 µM) were incubated with USP53^20-383^ (500 nM) or USP54^21-369^ (300 nM). Cleavage activity was analyzed by as shown in Fig. 3a-b. Uncropped versions of all gels and blots are provided as Source Data. **d**. Schematic of the identified cleavage activities of USP53 and USP54 on GFP modified with K63- or K48-linked triubiquitin chains. **e**. Overview of ubiquitin pull-down reagents used in this study. Data are derived from the original publications^52,54,55^. **f**. Deconvoluted intact protein mass spectra of biotinylated reagents. Calculated and observed molecular weights are given in Dalton. The species in the pan-polyUb TUBE denoted with an asterisk corresponds to a non-covalent acetonitrile adduct. **g-h**. MARVELD2 ubiquitination analysis through OtUBD pull-downs. Parental CaCo-2 cells (g) or CaCo-2 shRNA1 (USP53) cells (h) were analyzed as described in Fig. 3g. **i**. Input protein levels for samples used in Fig. 3g and panel j and analyzed as described for Fig. 3i. **j**. MARVELD2 polyubiquitination analysis with pan-polyubiquitin (4x UBA^UBQLN1^) TUBE pull-downs. **k**. UbiCRest assay on OtUBD eluates as in Fig. 3l, but with 2 h incubation. Source data

A strikingly different picture emerged for USP53, which preferentially cleaved polyubiquitin completely off GFP in a K63-linkage-dependent manner as evident from gel-based cleavage assays (Fig. 3a and Extended Data Fig. 4a–d). We followed the conversion of the GFP substrates using in-gel fluorescence and observed the emergence of free GFP. Coomassie staining of the same gel visualized free ubiquitin chains generated by USP53. Importantly, GFP–diubiquitin was converted into GFP and free diubiquitin (Fig. 3a). The earliest time points of the GFP–triubiquitin and GFP–tetraubiquitin assays showed the emergence of free triubiquitin and tetraubiquitin, respectably, which were later broken down into shorter chains consistent with the tetraubiquitin panel. The formation of free diubiquitin and triubiquitin chains was validated by intact protein mass spectrometry, which unequivocally confirmed the surprising en bloc deubiquitination activity of USP53 (Extended Data Fig. 4a-b). The emergence of low amounts of monoubiquitinated GFP in GFP–triubiquitin and GFP–tetraubiquitin assays demonstrates that substrate deubiquitination is not exclusive and cleavage in substrate-bound chains can take place in K63-linked chains longer than two ubiquitin moieties. Importantly, GFP conjugated to K48-linked diubiquitin or triubiquitin chains was not cleaved under identical conditions (Extended Data Fig. 4c,d), demonstrating linkage specificity of this activity.

Previously reported ubiquitin linkage-specific DUBs were described to operate strictly within ubiquitin chains. To our knowledge, an enzyme for which ubiquitin chains of a defined linkage direct deubiquitination activity for the preferred en bloc removal of this modification from substrates has not been described^2,14,16,17,20,45,46^. Our data strengthen the hypothesis that the en bloc deubiquitination activity of USP53 is facilitated by ubiquitin binding to both the S1 and S2 sites (Extended Data Fig. 4d)^20,45^. Taken together, these results show that USP53 and USP54 are active DUBs with intriguing K63 linkage and chain length specificities and that USP53 possesses unique K63-linkage-directed deubiquitination activity.

### Tricellular tight junction proteins are cellular substrates of USP53

To validate this activity in a cellular context and investigate possible consequences related to *USP53* loss, we generated stably transduced CaCo-2 cell lines carrying a doxycycline-inducible short hairpin RNA (shRNA) against *USP53*. CaCo-2 is a widely used human model cell line for the study of epithelial cell barriers and *USP53* shRNA induction led to effective depletion of the USP53 protein (Fig. 3c). We next assessed the effect of USP53 depletion on the ubiquitination status of proteins by ubiquitinome analysis, carried out by enrichment of diglycine-modified peptides and data-independent acquisition (DIA) mass spectrometry analysis (Fig. 3d)^3^. The top four ubiquitination sites, whose abundance increased upon *USP53* depletion, included K412 of MARVELD2 (also termed tricellulin) and K638 of LSR (also termed angulin-1) (Fig. 3e). Markedly, these two sites are within the cytoplasmic domains of MARVELD2 and LSR, for which genetic mutations in humans lead to the same phenotypes as *USP53* mutation (Fig. 3f)^36,47^. Both membrane-spanning proteins are core components of specialized cell–cell contact sites termed tricellular tight junctions^48^, with LSR recruiting MARVELD2 to tricellular tight junctions^49^, and are essential for epithelial barrier function^47,50,51^.

To corroborate ubiquitination, we used the high-affinity ubiquitin-binding domain OtUBD^52,53^ in a denaturing pulldown of cellular ubiquitin (Extended Data Fig. 4e,f). Blotting for MARVELD2 revealed its increased ubiquitination upon USP53 depletion (Fig. 3g). We observed the strongest change in a species whose molecular weight fits to MARVELD2 modified with two ubiquitin moieties, which we assigned as K63 diubiquitination on the basis ofour biochemical data (Figs. 1f and 3a and Extended Data Fig. 4c). The same change in MARVELD2 ubiquitination was observed using a cell line in which USP53 was depleted through a different shRNA sequence but was not observed in parental CaCo-2 cells upon treatment with doxycycline (Extended Data Fig. 4g,h).

To directly assess the regulated ubiquitin linkage type, while remaining at endogenous ubiquitin levels, we analyzed the same samples with tandem ubiquitin-binding entities (TUBEs) (Extended Data Fig. 4e,f)^54^. Their avidity-based mechanism entails that longer chains are enriched more efficiently than short chains. Pulldown of polyubiquitinated proteins with a high-affinity nonselective 4xUBA^UBQLN1^ reagent showed only a modest increase in overall polyubiquitinated MARVELD2 upon USP53 depletion (Extended Data Fig. 4i,j). However, enrichment with a K63-specific tUIM^Rap80^ reagent^55^ revealed a strong increase in K63-linked polyubiquitinated MARVELD2 (Fig. 3h). Importantly, global levels of MARVELD2 remained unchanged (Fig. 3i), which supports the notion that nondegradative ubiquitination is being regulated by USP53.

To test whether USP53 can deubiquitinate MARVELD2, we devised a workflow in which ubiquitinated proteins are enriched and then separated from the high-affinity OtUBD reagent by elution under acidic conditions. Subsequent elevation of the pH in the eluates and incubation with recombinant DUBs allowed UbiCRest assays^15^ (Fig. 3j). We observed that the nonspecific DUB USP2 removed all modifications on MARVELD2, confirming ubiquitination. The K63-specific DUB AMSH* but not the K48-specific DUB OTUB1* increased the amount of monoubiquitinated MARVELD2, consistent with cleavage within K63-linked chains (Fig. 3k). In contrast, incubation with USP53 but not USP54 led to an increase in free MARVELD2 and a concomitant decrease in the K63-linked diubiquitinated species, whereas the monoubiquitinated species remained unchanged (Fig. 3l and Extended Data Fig. 4k). These results are fully consistent with the biochemically identified K63-linkage-directed en bloc deubiquitination activity and, in sum, validate MARVELD2 as a cellular substrate of USP53.

### A covalent USP54~K63-linked diubiquitin–PA complex for crystallization

**Fig. 4:**
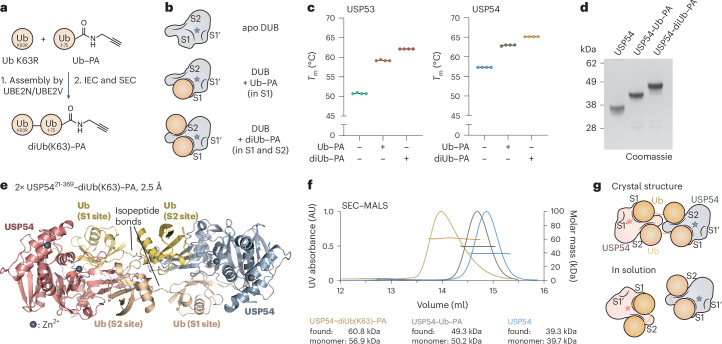
A K63-linked diubiquitin–PA probe enabled crystallization of USP54 in complex with ubiquitin. **a**, Schematic of the generation of a K63-linked diubiquitin probe, which was enzymatically assembled from ubiquitin K63R and ubiquitin–PA. IEC, ion-exchange chromatography. **b**, Schematic of catalytic DUB domains after reaction with ubiquitin–PA or diubiquitin–PA probes, illustrating ubiquitin engagement in samples used in **c**,**d**. S1′, S1 and S2 ubiquitin-binding sites are labeled. The active site cysteine is depicted as a star. **c**, Protein stability assessment. USP53^20–368^ and USP54^21–369^ were labeled with ubiquitin–PA or diubiquitin–PA probes and the stability of protein samples was analyzed by thermal shift analysis. Melting temperatures (*T*_m_) are shown for technical replicates, indicating the contribution of the S2 site. **d**, Purified DUB samples. USP54^21–369^ and USP54^21–369^ conjugated to ubiquitin–PA and USP54^21^^–^^369^ conjugated to K63-linked diubiquitin–PA were analyzed by SDS–PAGE and Coomassie staining. **e**, Crystal structure of USP54 conjugated to K63-linked diubiquitin–PA. In the observed complex, two USP54 molecules (blue and red) engage two K63-linked diubiquitin–PA molecules (gold and wheat) crosswise. The two isopeptide bonds between the diubiquitin–PA molecules are highlighted and are shown as sticks. **f**, SEC–MALS experiment of the catalytic domain of USP54^21–369^ alone (blue), in complex with ubiquitin–PA (brown) or in complex with K63-linked diubiquitin–PA (yellow), demonstrating monomeric species in solution. **g**, Schematic depiction of the organization of USP54 conjugated to K63-linked diubiquitin–PA in the crystal and in solution. Source data

**Extended Data Fig. 5:**
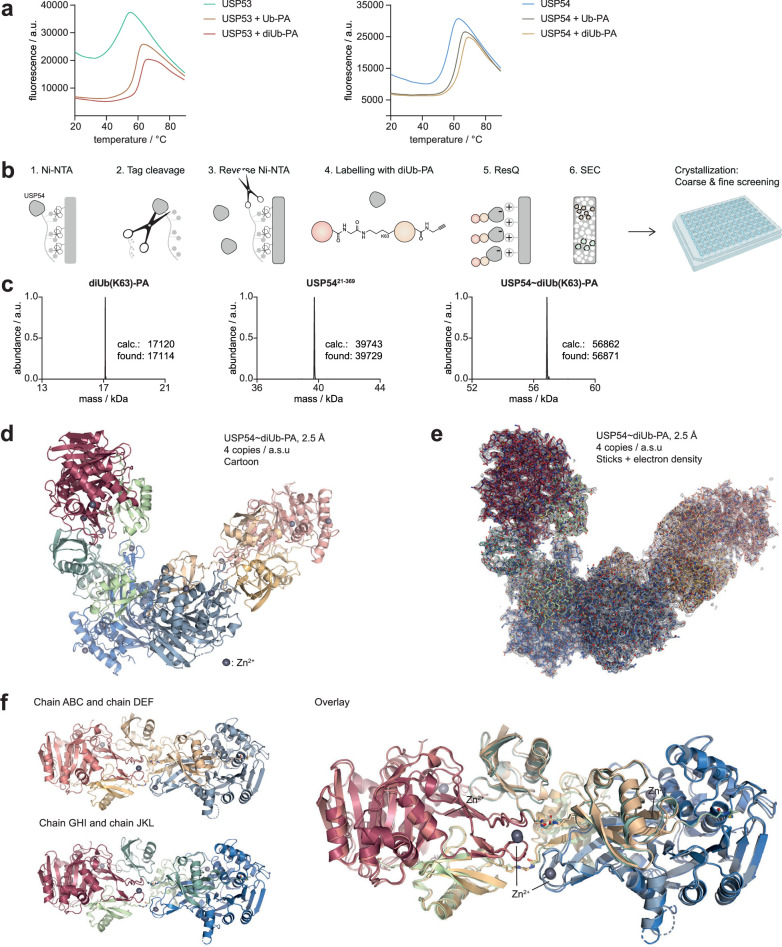
Assembly and crystallization of a USP54-diUb(K63)-PA complex. **a**. Fluorescence raw data of the protein stability assessment. Corresponding melting temperatures are shown in Fig. 4c. **b**. Assembly and purification strategy to obtain a pure USP54~diUb(K63)-PA complex for crystallization. Of note, cleavage of the native isopeptide bond in the probe was suppressed by protein labeling at low temperature and with an excess of probe over enzyme. **c**. Deconvoluted intact protein mass spectra of the K63-linked diUb-PA probe, USP54, and the USP54~diUb(K63)-PA complex. Calculated and observed molecular weights are given in Dalton. **d**. Asymmetric unit (a.s.u.) of the crystal structure of USP54~diUb-PA. All four copies of USP54~diUb-PA are shown in cartoon representation. **e**. Asymmetric unit (a.s.u.) of the crystal structure of USP54~diUb-PA as in panel d, all four copies are shown in stick representation overlayed with 2*F*_O_-*F*_C_ electron density contoured at 1 σ. **f**. Chains ABCDEF and chains GHIJKL, each consisting of 2x USP54~diUb-PA, are shown individually and as an overlay in cartoon representation. Source data

We next set out to investigate the mechanism of K63 selectivity and how these DUBs recognize chains. To visualize the additional S2 binding site, we aimed to solve a crystal structure of USP53 or USP54 in complex with K63-linked diubiquitin–PA. We prepared this isopeptide-linked probe by enzymatically assembling ubiquitin K63R onto ubiquitin–PA (Fig. 4a). A similarly designed K48-linked diubiquitin probe comprising a triazole linkage was previously used for the identification of a K48-specific S2 ubiquitin-binding site of a severe acute respiratory syndrome coronavirus (SARS-CoV) papain-like protease (PLpro) enzyme^16,56^. We used our probe alongside ubiquitin–PA to assess ubiquitin engagement in the prepared complexes (Fig. 4b). In thermal stability assays, diubiquitin–PA binding stabilized USP53 and USP54 more than ubiquitin–PA, indicating occupation of the S2 site (Fig. 4c and Extended Data Fig. 5a).

We prepared and purified several probe-conjugated DUB samples for extensive crystallization trials (Fig. 4d and Extended Data Fig. 5b,c). Following construct optimization, we obtained crystals for the most stable complex, USP54 conjugated to diubiquitin–PA (Fig. 4c,d), allowing the determination of a structure to 2.5-Å resolution by X-ray crystallography (Supplementary Table 2). Its asymmetric unit contained four copies of USP54 conjugated to diubiquitin–PA (Extended Data Fig. 5d,e), of which two copies formed an identical arrangement (Extended Data Fig. 5f). Excellent electron density covering the entire complex revealed that the two ubiquitin moieties of the probe engaged two different USP54 copies with symmetrical binding in *trans* configuration (Fig. 4e and Extended Data Fig. 5e). To assess the solution state of this complex, size-exclusion chromatography with multiangle light scattering (SEC–MALS) measurements were performed, proving that all USP54 samples are monomeric in solution (Fig. 4f). These results allowed the assignment of the dimeric arrangement of USP54 conjugated to diubiquitin–PA as a crystal artifact, likely derived from stabilization of the finger domain of USP54. We propose that, in solution, the complex of USP54 conjugated to diubiquitin–PA exists as a monomer and that USP54 engages K63-linked ubiquitin chains through its S1 and the S2 sites in *cis* configuration (Fig. 4g).

### USP54 contains a USP catalytic domain with a weakened S1 site

**Fig. 5:**
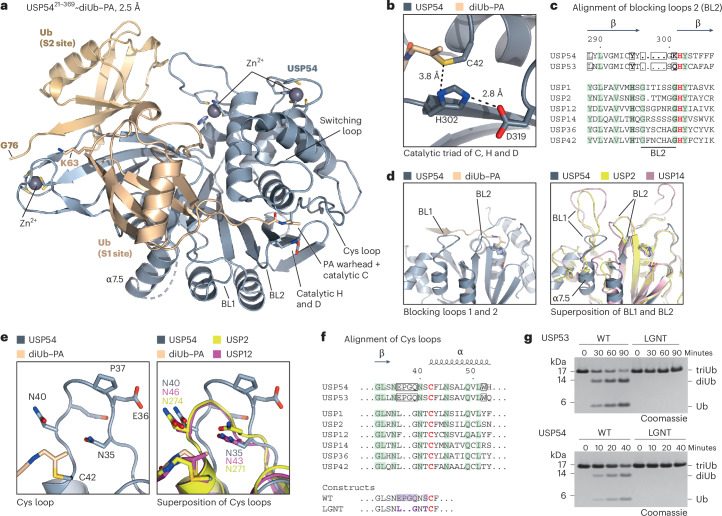
Catalytic activity of USP53 and USP54 depends on a unique Cys loop. **a**, Solution arrangement of USP54 conjugated to K63-linked diubiquitin–PA. Cartoon representation depicting USP54^21–369^ (gray) with ubiquitin moieties bound in its S1 (wheat) and S2 (gold) binding sites. Zinc atoms are shown as gray spheres. **b**, Close-up view of the catalytic triad. Indicated distances are visualized by dotted lines. **c**, Sequence alignment of residues forming BL2 in USP DUBs. Catalytic histidines are colored red, while conserved residues are colored green. Residues or gaps in USP54 and USP53, which are unique across the entire human USP family, are highlighted with a box. Residues strongly conserved in the USP DUB family, which led to the initial mischaracterization of USP53 and USP54 as inactive, are highlighted in bold. Secondary-structure elements and numbering are according to USP54. **d**, Left, close-up view of BL1 and BL2 of USP54. Right, superposition with the corresponding residues in USP2 (PDB 2IBI, yellow) and USP14 (PDB 2AYO, purple), illustrating the atypical shortening of both loops in USP54. **e**, Left, close-up view of the unique loop of USP54 close to the catalytic cysteine. Right, superposition of the Cys loop with the corresponding residues in USP2 (yellow) and USP12 (PDB 5L8W, magenta). **f**, Sequence alignment of residues forming the Cys loop in USP DUBs (annotation as in **c**). Sequences of USP53 and USP54 constructs used to study the Cys loop are shown below with changes marked in violet. **g**, Gel-based ubiquitin chain cleavage assay. K63-linked triubiquitin chains (3 µM) were incubated with USP53^20–383^ (3 µM) or USP54^21–369^ (300 nM) as either WT or Cys loop mutant (labeled as LGNT) (sequences in **f**) for the indicated time points. Cleavage activity was analyzed by SDS–PAGE and Coomassie staining. Source data

**Extended Data Fig. 6:**
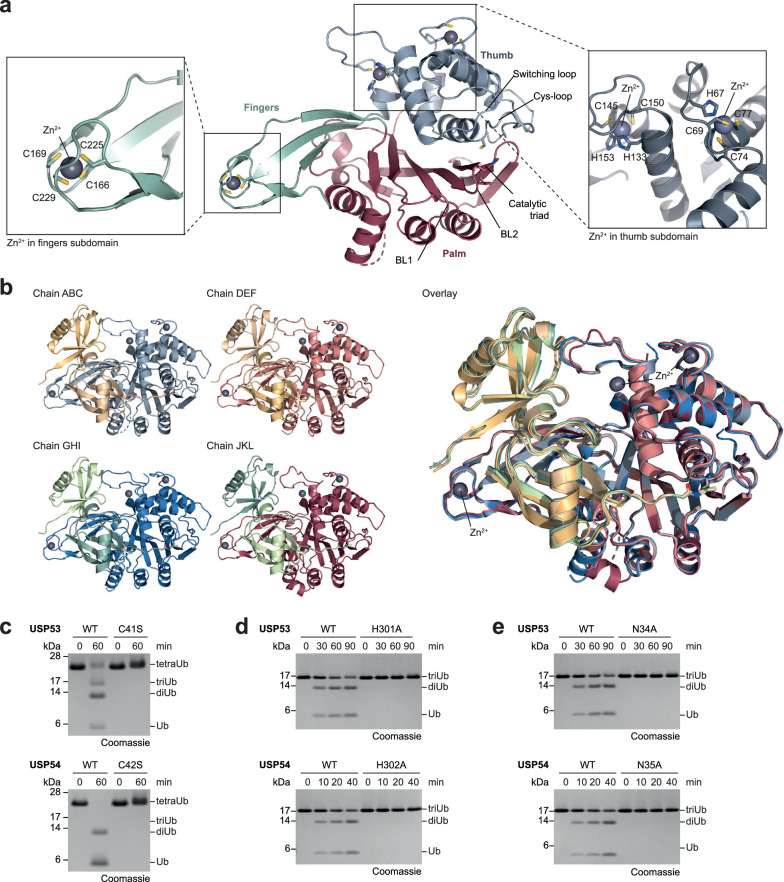
Analysis of the USP fold in the USP54~diUb-PA structure. **a**. Typical fold of USP DUBs in the structure of USP54~diUb-PA. The palm subdomain is colored red, the thumb subdomain blue and the fingers subdomain green. Additional structural elements including the blocking loops, the switching loop, the Cys loop, the zinc ions, and the catalytic triad are annotated. Close-up views for the coordination of all zinc ions are shown. **b**. Chains ABC, DEF, GHI and JKL of the crystal structure of USP54~diUb-PA, corresponding to the conformation in solution, are shown individually and as an overlay in cartoon representation. **c**. Gel-based cleavage assay assessing the catalytic cysteines. K63-linked tetraubiquitin chains (2 µM) were incubated with USP53^20-383^ (4 µM, *upper*) or USP54^21-369^ (300 nM, *lower*) and the respective catalytic cysteine mutants. Cleavage activity was analyzed by SDS-PAGE and Coomassie staining. **d**. Gel-based cleavage assay assessing the catalytic histidines. K63-linked triubiquitin chains (3 µM) were incubated with USP53^20-383^ (2 µM, *upper*) or USP54^21-369^ (300 nM, *lower*) and the respective catalytic histidine mutants for indicated time points. **e**. Gel-based cleavage assay as in d assessing potential oxy-anion hole residues. Uncropped versions of all gels are provided as Source Data. Source data

USP54 adopts the USP DUB catalytic domain fold consisting of hand, thumb and finger subdomains (Extended Data Fig. 6a). A model for the solution state illustrated binding of the distal ubiquitin to an S2 site located at the back of the USP fingers subdomain (Fig. 5a). An identical orientation was observed for all four USP54 molecules within the asymmetric unit (Extended Data Fig. 6b). Moreover, we found the C-terminal residues of the ubiquitin in the S2 site to be in close proximity to the K63 side chain of the S1 ubiquitin (Fig. 5a). This is consistent with an in-solution structure in which the isopeptide bond is placed on top of the fingers without the need for reorientation of the ubiquitin moieties.

We next analyzed the USP54 catalytic domain and its engagement of the S1 ubiquitin. A canonical catalytic triad was positioned in an active conformation, with the warhead of the diubiquitin–PA probe covalently linked to the catalytic cysteine (Fig. 5b). The cleavage activity on K63-linked ubiquitin chains was abolished for catalytic cysteine and histidine mutants (Extended Data Fig. 6c,d). Moreover, the structure revealed three coordinated zinc ions, which comprised two positions within the thumb subdomain in addition to the common position at the tip of the fingers (Extended Data Fig. 6a). One of these was identified in human USP36 (ref. ^41^), whereas coordinating residues for the second zinc are not present in other human USP DUBs^32^. The positions of the three ions loosely resemble those in the USP domain within the yeast SAGA DUB module^57,58^.

**Extended Data Fig. 7:**
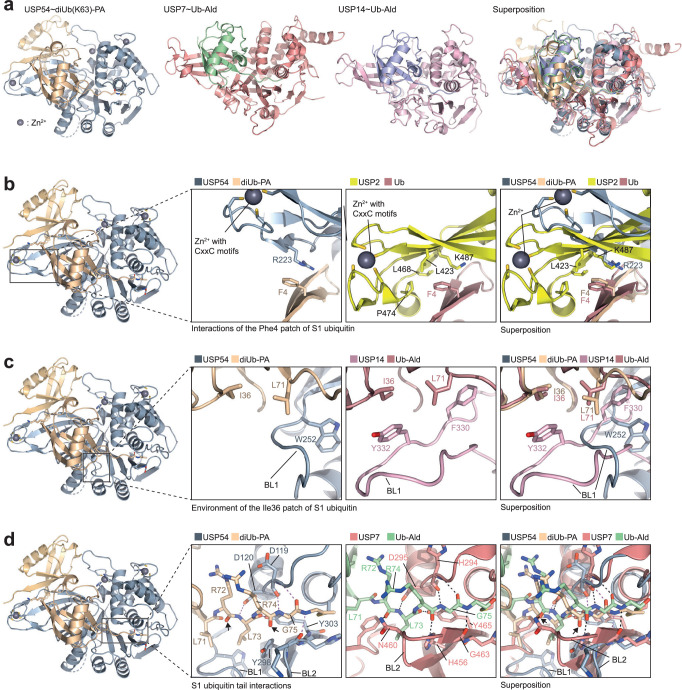
Analysis of the weakened S1 ubiquitin recognition in USP54. **a**. Cartoon representation of USP54~diUb-PA as well as of representative USP DUB-ubiquitin aldehyde complexes (USP7, PDB: 1NBF; USP14, PDB: 2AYO). A superposition is shown, illustrating the distinct relative geometry of ubiquitin in the S1 site of USP54. **b**. Close-up view on the interaction of the Phe4 patch in the S1 ubiquitin with USP54 (*left*) and USP2 (PDB: 2IBI, *center*). A superposition (*right*) (aligned on ubiquitin) illustrates the different environment of Phe4 in USP54. **c**. Close-up view on the interactions of BL1 in USP54 (*left*) and in USP14 (PDB: 2AYO, *center)* with the S1 ubiquitin. A superposition (*right*) based on the S1 ubiquitin is shown. **d**. Close-up view on the S1 ubiquitin C-terminal tail interactions with USP54 (*left*) and USP7 (PDB: 1NBF, *center*). Hydrogen bonds are illustrated by dotted lines in pink for USP54 and black for USP7. Arrows highlight the missing hydrogen bonds in USP54 in comparison to USP7, also visible in the superposition (*right*).

Engagement of the S1 ubiquitin by USP DUBs typically relies on (1) hydrophobic contacts to the F4 patch by the frontside of the finger subdomain; (2) a hydrophobic contact of the I36 patch by blocking loop 1 (BL1); and (3) guiding of the ubiquitin C-terminal residues through the active site cleft by blocking loop 2 (BL2)^41,59^. In USP54, the S1 ubiquitin displayed a ~30° rotation in comparison to the common binding mode (Extended Data Fig. 7a) and differed in all three main interaction sites. Firstly, the hydrophobic residues normally contacting F4 were missing, leaving this patch largely solvent exposed (Extended Data Fig. 7b). Secondly, I36 of ubiquitin was solvent exposed (Extended Data Fig. 7c), as BL1 appeared absent, with corresponding residues adopting a unique helix (α7.5) oriented away from ubiquitin. This arrangement has not previously been observed for USP DUBs (Fig. 5c,d). Thirdly, BL2 of USP54 was truncated (Fig. 5c,d), leaving amide bonds in the ubiquitin C-terminal residues without the otherwise occurring zip-like hydrogen bonding (Extended Data Fig. 7d). Collectively, these differences, which appear to be shared by USP53 and USP54 according to the primary sequence, suggest a weakened S1 ubiquitin-binding site. This is consistent with the comparably weak activity of both DUBs on monoubiquitin substrates (Extended Data Fig. 1f and Fig. 2g).

Another feature of USP53 and USP54, which is unique among human USP DUBs, is an elongated Cys loop directly before the catalytic cysteine (Fig. 5e,f). The additional residues are flanked by two asparagine residues conserved in the USP family^32^, of which N35 acts as an oxyanion hole. Substitution of this residue in both USP53 and USP54 verified its importance for catalytic activity (Extended Data Fig. 6e). Moreover, amino acid changes in the unique Cys loop sequence (EPGQNSC) of both DUBs into the commonly observed sequence (LGNTC) completely abolished cleavage of K63-linked chains (Fig. 5g).

**Extended Data Fig. 8:**
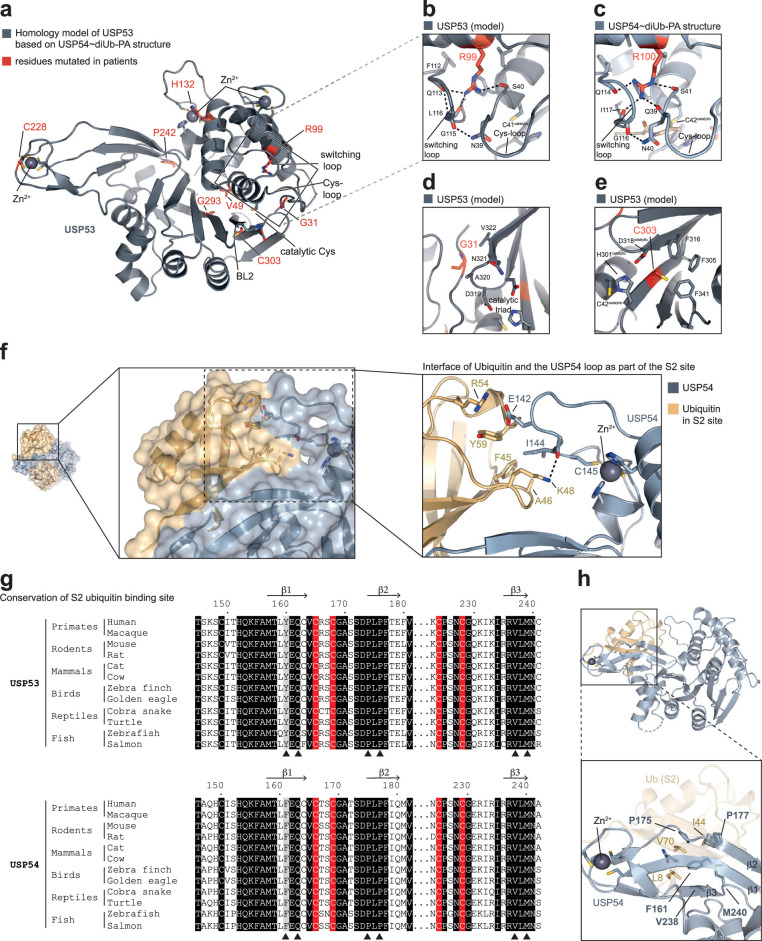
Structural analysis of cholestasis-linked mutations in USP53 and conservation of S2 site on back of USP fingers in USP53 and USP54 orthologues. **a**. Homology model of the USP53 catalytic domain, based on the USP54 crystal structure. Residues of disease-associated single amino acid mutations are shown as red sticks (see Fig. 2a). **b**. Close-up view on Arg99 in USP53, highlighting bridging interactions to the switching loop and the Cys loop. **c**. Close-up view on the corresponding residue Arg100 of USP54 in the USP54~diUb(K63)-PA structure. **d-e**. Close-up view on Gly31 and Cys303 in the USP53 model, highlighting how substitution of these closely surrounded residues for amino acids with larger side chains in the patient mutations G31S and C303Y likely lead to structural perturbations very close to catalytic residues. **f**. Close-up views on the S2 site, highlighting how a loop in the USP54 thumb domain above the S2 site forms engages ubiquitin. **g**. Sequence alignment of representative USP53 and USP54 orthologues showing the first three USP fingers beta sheets. Secondary structures and numbers are indicated according to human USP54. Species include human (*Homo sapiens*), Macaque monkey (*Macaca mulatta*), mouse (*Mus musculus*), rat (*Rattus norvegensis*), cat (*Felix catus*), cow (*Bos taurus*), zebra finch (*Taeniopygia guttata*), golden eagle (*Aquila chrysaetos chrysaetos*), Indian cobra (*Naja naja*), painted turtle (*Chrysemys picta bellii*), zebrafish (*Danio rerio*) and atlantic salmon (*Salmo salar*). Residues conserved in all sequences are shown in black, the S2 aromatic residues are shown in grey. Cystein residues coordinating the zinc ion at the tip of the fingers are highlighted in red. Residues on the beta sheets with side chains pointing towards the backside / S2 ubiquitin binding site are highlighted with a black triangle. **h**. Coordination of the Ile44 patch in the S2 ubiquitin binding site. Cartoon representation of the USP54~diUb-PA structure. Ubiquitin residues of the Ile44 patch and residues highlighted with a black triangle in panel g are labeled as sticks.

Mapping all cholestasis-related single amino acid substitutions in USP53 (Fig. 2a) on a homology model based on our USP54 structure illustrated that several substituted residues are either part of the above-described zinc fingers (explaining the reduced stability and impaired catalytic activity of the H132Y protein) or are located around the active site (Extended Data Fig. 8a–e). The model indicates how G31S and C303T lead to structural perturbations in immediate vicinity of the catalytic residues (Extended Data Fig. 8d,e), explaining the impaired catalytic activity of USP53 proteins with these substitutions (Fig. 2c,d). A closer inspection of R99 in USP53 or of the equivalent residue R100 in the USP54 structure revealed critical roles in coordinating the unique Cys loop to the switching loop (Extended Data Fig. 8b,c). Taken together, these data demonstrate the importance of the unique active site arrangement in both USP DUBs for catalytic activity and enable the structural rationalization of *USP53* mutations associated with disease (Supplementary Table 1).

### A cryptic S2 site within the USP fingers engages K63-linked chains

**Fig. 6:**
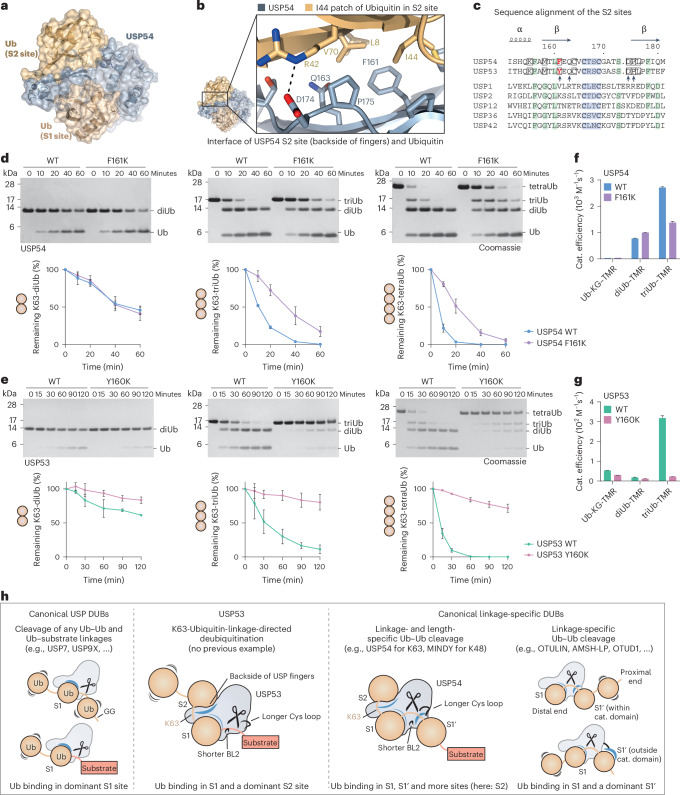
A cryptic S2 ubiquitin-binding site in USP53 and USP54 mediates efficient cleavage of K63-linked ubiquitin chains. **a**, Structure of USP54 conjugated to K63-linked diubiquitin–PA. **b**, Interface of ubiquitin and USP54 S2 site. **c**, Sequence alignment of residues forming the S2 site in USP54 and USP53. Cys-x-x-Cys motifs at the tip of the fingers (blue), conserved residues (green), residues unique in USP53 and USP54 within the human USP family (box) and residues annotated in **b** (arrows) are highlighted. **d**, Gel-based polyubiquitin cleavage assays. K63-linked ubiquitin chains (2 µM) were incubated with WT USP54^21–369^ or the S2 site mutant F161K (both at 300 nM). Substrate consumption was quantified by densitometry, normalized to initial intensities. Data are shown as the average ± s.d. of three independent replicates. **e**, Gel-based polyubiquitin cleavage assays of USP53, shown as in **d**. WT USP53^20–383^ or the S2 site mutant Y160K was used (2 µM). **f**, Catalytic efficiencies of USP54 proteins obtained from fluorescence polarization assays (substrates shown in Fig. 2d). Raw data are shown in Extended Data Fig. 9d,e and the catalytic efficiencies of WT protein are repeated from Fig. 2g. Data are shown as the mean ± s.e.m. **g**, Catalytic efficiencies of USP53 proteins, analyzed as in **f**. Efficient catalysis of USP53 is dependent on its S2 site. **h**, Schematic of ubiquitin processing by DUBs. K63-linkage-directed deubiquitination by USP53 bridges canonical DUB categories. The structural mechanisms for polyubiquitin length and linkage specificity in DUBs are shown in Extended Data Fig. 10. Source data

The structure of USP54 conjugated to diubiquitin–PA uncovered an S2 ubiquitin-binding site within its USP domain (Figs. 5a and 6a and Extended Data Fig. 8f), consistent with biochemical assay data. This interface is centered on the backside of the fingers subdomain and is governed by hydrophobic interactions with the I44 patch of ubiquitin. This main protein interaction site of ubiquitin comprises I44 in addition to L8, V70 and H68 (ref. ^1^). The central residue in USP54 at the contact site is F161 (Fig. 6b), which corresponds to Y160 in USP53, suggesting similar ubiquitin engagement. Notably, these hydrophobic residues on the backside of the USP fingers domain are strictly conserved in USP53 and USP54 orthologs (Extended Data Fig. 8g,h), whereas other human USP DUBs have varying and typically polar residues at these positions (Fig. 6c).

**Extended Data Fig. 9:**
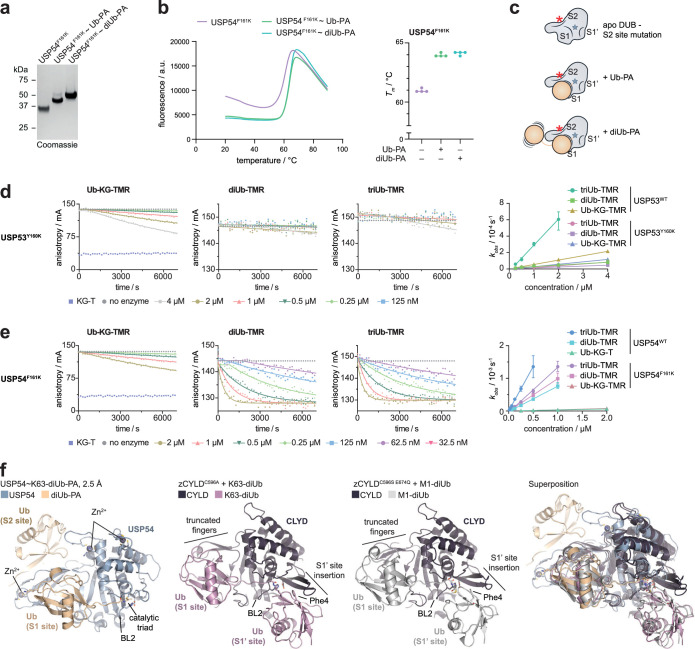
A cryptic S2 site mediates catalytic activity on longer ubiquitin chains. **a**. Purified samples of USP54 with mutated S2 site. USP54^21-369^ F161K, USP54^21-369^ F161K~Ub-PA and USP54^21-369^ F161K~diUb(K63)-PA were analyzed by SDS-PAGE and Coomassie staining. An uncropped version is provided as Source Data. **b**. Protein stability assessment of S2 site-mutated USP54. Stability of samples from panel **a** was analyzed by thermal shift analysis. Fluorescence raw data are shown. Melting temperatures (*T*_*m*_) are plotted from technical replicates. **c**. Schematic of catalytic USP54 F161K DUB domains after reaction with Ub-PA or diUb-PA probes, illustrating ubiquitin engagement in samples used in panels **a** and **b**. S1’, S1 and S2 Ub binding sites are labelled. The mutated S2 site is depicted as a red star. **d**. Fluorescence polarization cleavage assays for USP53^20-383^ Y160K and indicated reagents. Fluorescence anisotropy was recorded over time and observed rate constants (*k*_*obs*_, shown as mean ± standard error) were plotted over enzyme concentrations. Values for WT protein were taken from Fig. 2g. **e**. Fluorescence polarization cleavage assays for USP54^21-369^ F161K and indicated reagents as described in panel d. **f**. Cartoon representation of USP54~diUb-PA, CYLD in complex with K63-linked diUb (PDB: 3WXG) and CYLD in complex with M1-linked diUb (PDB: 3WXF). A superposition illustrates features enabling K63-linkage recognition in both enzymes by distinct mechanisms. The catalytic domain of CYLD achieves dual K63 and M1 polyubiquitin cleavage specificity through a unique β-hairpin inserted into its catalytic domain. This insertion forms an S1’ ubiquitin binding site to recognize the Phe4 patch of the proximal ubiquitin (in both K63- and M1-linked chains) and positions the substrate for efficient catalysis. USP54 lacks an equivalent hairpin insertion. Source data

We next sought to assess the importance of the S2 site for cleavage activity on K63-linked ubiquitin chains. An F161K substitution abrogated binding of the distal ubiquitin through disruption of the S2 site (Extended Data Fig. 9a–c). This substitution led to a decrease in cleavage of triubiquitin and tetraubiquitin chains by USP54 (Fig. 6d), visualized through quantitative gel-based cleavage assays. Importantly, the substitution did not influence the cleavage of K63-linked diubiquitin, demonstrating that binding of ubiquitin chains to the S1 and S1′ sites is not affected by a change in the S2 site (Fig. 6d). Strikingly, an equivalent Y160K substitution in USP53 drastically reduced the cleavage of triubiquitin and tetraubiquitin chains (Fig. 6e), indicating that efficient catalysis for USP53 is driven through the S2 site.

To confirm these findings, we assessed both mutant proteins in fluorescence polarization assays (Fig. 6f,g and Extended Data Fig. 9d,e). Consistently, amino acid changes impacting the S2 sites of USP53 or USP54 did not change the catalytic activities on monoubiquitin and diubiquitin substrates. However, triubiquitin cleavage activities were dampened. USP53 Y160K showed cleavage activity barely above background (Fig. 6g), proving the critical importance of the S2 site for catalysis. The more dramatic effect of the amino acid changes in USP53 compared to USP54 is consistent with the higher activity of USP54 on diubiquitin substrates.

Collectively, these data demonstrate that both USP53 and USP54 possess an additional K63-linkage-specific S2 ubiquitin-binding site embedded into their USP catalytic domains. Engagement of the ubiquitin I44 patch by the back of their fingers subdomains is unique within the USP family of DUBs and mechanistically explains their preference for K63-linked ubiquitin chains.

## Discussion

We here revise the annotation of USP53 and USP54 from inactive pseudo-DUBs^32^ to active DUBs and report in USP53 the discovery of K63 ubiquitin linkage-directed deubiquitination activity. This unprecedented mode of human DUB activity expands the previously established categories of canonical USP DUBs, which typically cleave isopeptide linkages promiscuously, and of linkage-specific DUBs, which edit chains but do not deubiquitinate substrates (Fig. 6h)^20^. In USP53, recognition of K63-linked chains through an S2 site embedded within its catalytic domain is a prerequisite for efficient catalysis. USP53 shares with its homolog USP54 a weakened S1 ubiquitin-binding site through truncations in BL1 and BL2, which explain their comparably poor reactivity on monoubiquitin substrates.

**Extended Data Fig. 10:**
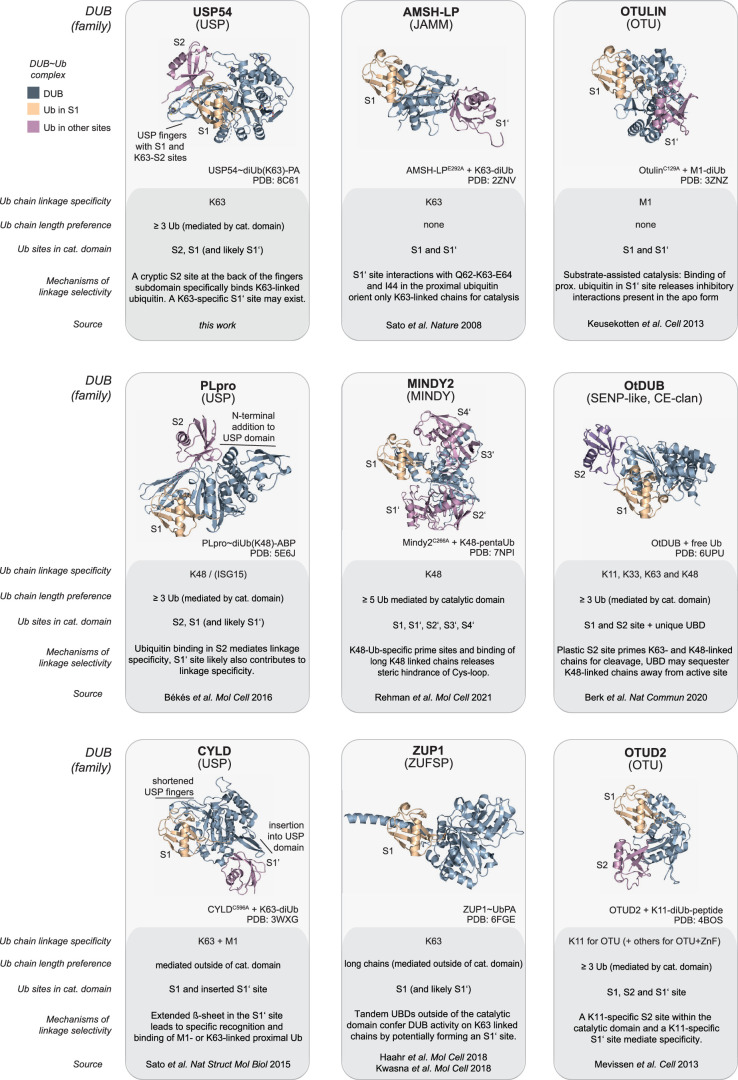
Mechanisms for polyubiquitin length- and linkage-specificity in DUBs.

Catalysis driven by an S2 ubiquitin-binding site has previously been reported for PLpro enzymes of SARS-CoV^16^ and SARS-CoV-2 (ref. ^17^), albeit with K48 linkage specificity. These enzymes possess N-terminal extensions to their USP-like catalytic domain to recognize ISG15 and K48-linked polyubiquitin (Extended Data Fig. 10), for which hydrophobic recognition of the distal ubiquitin is critical^60^. While K48-linkage-directed substrate deubiquitination by these enzymes has been evaluated in vitro^45,60^, evidence for this activity on cellular substrates is currently not available. Structural^16^ and biochemical^45^ studies on SARS-CoV PLpro concluded that cleavage within K48-linked polyubiquitin is preferred over K48-linkage-directed substrate deubiquitination. This is in contrast to USP53, which, when evaluated with purified substrate-bound ubiquitin chains, preferred the en bloc removal of K63-linked polyubiquitin over cleavage within the chain (Fig. 3a and Extended Data Fig. 4a,b). Moreover, cellular investigation revealed changes in K63-linked ubiquitination of MARVELD2, the emergence of a diubiquitin-modified protein and changes in ubiquitination site abundance in MARVDEL2 and LSR upon *USP53* deletion (Fig. 3). These findings are consistent with the unique activity we characterize and, together, set it apart from canonical linkage-specific DUBs. Importantly, deubiquitination by USP53 is greatly accelerated by K63-linked but not by K48-linked polyubiquitin, as expected from the structure-guided biochemical analysis of its K63-specific S2 site (Fig. 6). At high concentrations or long incubation times, low cleavage activity on other linkage types can be detected for USP53. The same has also been observed for the M1/K63-specific enzyme CYLD^42^ and is in line with the typically broad catalytic scope of USP family members. This explains why linkage-directed deubiquitination activity could emerge in a USP-fold enzyme with weakened S1 sites and an added S2 site. Investigation of a potential S1′ site in future work could illuminate molecular determinants for en bloc substrate deubiquitination versus cleavage within chains.

En bloc removal of ubiquitin chains from substrates has previously been demonstrated for proteasome-resident DUBs PSMD14 and USP14, which is independent of the chain linkage type^20,46,61^. Notably, the mechanism of K63-linkage-directed deubiquitination of USP53 is also distinct from the established activity of USP5 (ref. ^62^), which drives efficient cleavage of free ubiquitin chains through an S1′ site outside of its catalytic domain and irrespective of the linkage type. Thus, the mechanism of USP53 functionally expands the described DUB activities in human cells.

USP54 is also an atypical USP DUB family member with K63 specificity and a preference for longer chains. Preferred cleavage of longer chains has been reported for (1) ZUFSP for K63 linkages^6,11,23,25^, with a yet unknown mechanism; (2) MINDY DUBs with K48-specific S2′ through S4′ ubiquitin sites^14,22^; (3) the SARS-CoV PLpro enzyme with a K48-specific S2 site in the N-terminal extension of its catalytic domain^16,17^; and (4) OTUD2 with a K11-specific S2 site^15^. The mode of ubiquitin chain recognition by USP54 is distinct from all abovementioned examples and from the K63-specific DUBs AMSH^26^ and CYLD^42^ (Extended Data Fig. 9f), which contain S1′ but not S2 sites within their catalytic domains (Extended Data Fig. 10). While K63-linked chains of 2–4 ubiquitin moieties exist on substrate proteins in cells^7^ and can be recognized in a length-dependent manner^12^, how K63 polyubiquitin chain length can be decoded by DUBs on the molecular level was previously not known. These findings will guide the assessment of ubiquitin-dependent roles of cellular, full-length USP54 and stimulate research into the chain-length-dependent fine-tuning of ubiquitin signaling.

Mutations in *USP53* have clinically been related to bile acid transport disorders, a subgroup of cholestasis^63^ also comprising cases with mutations in *TJP2* (tight junction protein 2)^64^ and in *LSR*^36^. Some persons with congenital *USP53* alterations also presented with hearing loss^34,37^, consistent with the mouse *USP53* phenotype^30^. Notably, deafness is also caused by mutations in genes encoding cell–cell junction proteins including claudins, *ILDR1* and the MARVELD family members occludin and tricellulin (*MARVELD2*)^47,51^. USP53 was shown to localize to cell–cell barriers in mouse inner ear cells and to interact with TJP1 and TJP2 (ref. ^30^). Consistent with our biochemical and structural data and the proposed model for USP53 activity, we found elevated ubiquitination on the two core components of tricellular junctions MARVELD2 and LSR upon knockdown of *USP53*. The combined use of diglycine proteomics, different ubiquitin enrichment techniques and UbiCRest-like assays validates these as USP53 substrates and synergizes with recent advances in the assessment of ubiquitin chain architecture^3,7,65^ These substrates are supported by coinciding phenotypes of deafness and cholestasis^36,47^, consistent with the *USP53* mouse phenotype^30,47^. Collectively, the results converge into a model in which loss of the USP53 catalytic activity reported herein causes pediatric cholestasis (Fig. 3).

Our work critically guides the investigation of ubiquitin-dependent cellular roles of USP53 and USP54. Our structural insights will aid the molecular investigation of both enzymes and provide a framework for the understanding of *USP53* disease-causing mutations. Moreover, our results demonstrate how ubiquitin chains can lead to en bloc protein deubiquitination by a human DUB in a chain-linkage-dependent manner. This unique activity enables the deubiquitination of polyubiquitinated proteins while preserving monoubiquitin modifications on the same substrate. Lastly, our data stimulate the re-evaluation of known DUBs with isopeptide-linked polyubiquitin substrates, which represent endogenous structures more closely and complement frequently used panels of free ubiquitin chains, in search for perhaps other enzymes featuring linkage-directed deubiquitination activity.

## Methods

### Cloning and constructs

Catalytic domain constructs of human USP53 (residues 20–368 and 20–383; UniProt Q70EK8) and USP54 (residues 21–369 and 21–385; UniProt Q70EL1) were cloned from *Escherichia coli* codon-optimized DNA into the pOPINK vector for bacterial expression (N-terminal His_6_-GST-3C-tag) with In-Fusion cloning (Takara Clonetech). Site-directed mutagenesis was carried out through PCR according to the QuikChange protocol using Phusion polymerase (New England BioLabs (NEB)).

DNA sequences coding for human ubiquitin (Uniprot P0CG47) or human Ubls SUMO1, SUMO2, SUMO4, SUMO5, ISG15, UFM1, FUBI, URM1 and DWNN (UniProt P63165, P61956, Q6EEV6, G2XKQ0, P05161, P61960, P62861, Q9BTM9 and Q7Z6E9, respectively) were cloned into the pTXB1 vector (NEB) with N-terminal HA tags as described previously^41^. Ubiquitin and ubiquitin variants were expressed from pET17b.

Constructs for mouse E1 (mE1), UBE2R1, UBE2V1, UBE2N, UBE2S-UBD, NleL, UBE2L3/UbcH7, AMSH* and OTUB1* were used as described previously^66^. UBE2S and UBE2N were cloned from plasmids encoding UBE2S-UBD and UBE2N into pOPINK.

Enhanced monomeric GFP was cloned from a previously described vector^67^ with a C-terminal LACE tag (GSGPRKVIKMESEE) (GFP-C-LACE-tag) into pOPINK.

To obtain shRNA-encoding vectors, the Tet-pLKO-puro plasmid was sequentially digested by AgeI and EcoRI followed by ligation with annealed sense and antisense shRNA oligonucleotides using T4 DNA Ligase (NEB). The following sequences were used:

shRNA1 (*USP53*), forward: CCGGGGTCACATTGATATGAAATATCTCGAGATATTTCATATCAATGTGACCTTTTTG

shRNA1 (*USP53*), reverse: AATTCAAAAAGGTCACATTGATATGAAATATCTCGAGATATTTCATATCAATGTGACC

shRNA5 (*USP53*), forward: CCGGCACTTCCCTCAGATAACATAACTCGAGTTATGTTATCTGAGGGAAGTGTTTTTG

shRNA5 (*USP53*), reverse: AATTCAAAAACACTTCCCTCAGATAACATAACTCGAGTTATGTTATCTGAGGGAAGTG

shRNA nontargeting control (NTC), forward: CCGGAGTGAACGAGTAAGATAACCCTCGAGGGTTATCTTACTCGTTCACTTTTTTG

shRNA NTC, reverse: AATTCAAAAAAGTGAACGAGTAAGATAACCCTCGAGGGTTATCTTACTCGTTCACT

Constructs for ubiquitin enrichment reagents were cloned into pOPINB adding N-terminal Avi tags for the pan-polyubiquitin–TUBE (4xUBA^UBQLN1^) and the K63-linked polyubiquitin–TUBE (tUIM^Rap80^/Rx3A7) and an N-terminal cysteine for OtUBD.

### Protein expression

Rosetta 2(DE3)pLacI cells were used for bacterial protein expressions after transformation with the respective plasmids (apart from ubiquitin enrichment proteins, which were expressed in BL21(DE3) cells). The 2xTY medium supplemented with appropriate antibiotics was inoculated in a 1:100 ratio from an overnight culture and cells were grown shaking at 37 °C until an optical density of 0.7–1.0 was reached. After cooling to 18 °C, protein expression was induced by the addition of 0.5 mM IPTG and conducted overnight at 18 °C. Harvested bacterial cells were frozen and stored at −80 °C until further use.

### Protein purification

USP53 and USP54 were purified by Ni-NTA affinity purification, reverse Ni-NTA affinity purification, anion-exchange chromatography and SEC. Bacterial cells were resuspended in buffer N (20 mM NaH_2_PO_4_ pH 8.0, 300 mM NaCl, 20 mM imidazole, 4 mM β-mercaptoethanol (β-ME) supplemented with DNAseI, lysozyme, EDTA-free protease inhibitor cocktail (one tablet in 100 ml, Roche) and 1 mM PMSF) and lysed by sonication (55% amplitude; 5 s on, 10 s off) on ice. After pelleting, the lysate was loaded onto a Ni-NTA FF column (Cytiva) and eluted with buffer N containing 500 mM imidazole. Protein-containing fractions were pooled, His-3C protease was added and the samples were dialyzed overnight against buffer N. After reverse Ni-NTA, the flowthrough was dialyzed overnight against LS buffer (25 mM Tris pH 8.5, 50 mM NaCl and 4 mM β-ME) and subjected to anion-exchange chromatography using a Resource Q (ResQ) column (GE Healthcare) with an elution gradient ranging from 50 to 500 mM NaCl in LS buffer. Peaks containing the protein of interest were pooled, concentrated and either subjected to SEC using buffer S (20 mM Tris pH 8.0, 100 mM NaCl and 4 mM β-ME) on a HiLoad 16/600 Superdex 75pg column (GE Healthcare) or buffer-exchanged into buffer S.

Initially, a USP54 construct spanning amino acids 21–385 was expressed, which was found to be C-terminally truncated during purification. The mass of the main species matched to amino acids 21–369. A corresponding construct was generated and used for all subsequent experiments as indicated.

Purification of probe-labeled USP54 was performed similar to apo USP54. After Ni-NTA and reverse Ni-NTA purification, the protein solution was buffer-exchanged into 20 mM Tris pH 8.0, 100 mM NaCl and 5 mM DTT with the probe added in a 1:3 molar ratio and incubated overnight at 4 °C. A pure complex of USP54 conjugated to diubiquitin–PA was obtained from anion-exchange chromatography and SEC, which were performed as described for unlabeled USP54 (Extended Data Fig. 5).

OtUBD and Avi-tagged TUBE reagents were purified by Ni-NTA and SEC as described above with the adjustments of 50 mM NaH_2_PO4 in buffer N, a different SEC buffer (described below) and no protease inhibitor cocktail.

GFP-C-LACE-tag, UBE2N and UBE2S were purified by Ni-NTA purification, reverse Ni-NTA purification and SEC as described above for USP53. Purification protocols for GST fusion proteins of UBE2R, UBE2V, UBE2S-UBD, UBE2L/UBCH7 and NleL^68,69^, as well as for AMSH* and OTUB1*^70^, were adopted from the literature. mE1 was purified by Ni-NTA, ResQ and SEC. His-3C protease was obtained from Dortmund Protein Facility.

Ubiquitin, ubiquitin variants and M1-linked ubiquitin chains were purified by acidic precipitation, cation-exchange chromatography and SEC^71^. Cell pellets were resuspended in 20 mM Tris pH 7.4 and 2 mM EDTA and lysed by sonication (55% amplitude; 10 s on, 10 s off) on ice. The cleared lysate was mixed with 0.5% (v/v) perchloric acid on ice to induce protein precipitation. The remaining soluble protein was dialyzed against buffer F (50 mM sodium acetate pH 4.5) and subjected to cation-exchange chromatography using a HiPrep SP FF 16/10 column (GE Healthcare) with an elution gradient from 0 to 1,000 mM NaCl in buffer F. Fractions containing ubiquitin were further purified on a HiLoad 16/600 Superdex 75pg column in PBS.

Protein concentrations were determined by absorption measurements at 280 nm (Nanodrop) using calculated extinction coefficients and molecular weights. Aliquoted protein samples were snap-frozen in liquid nitrogen and stored at −80 °C until further use.

### Generation of ubiquitin-based and Ubl-based probes

Ubiquitin and Ubl probes with PA and VS warheads were prepared as described previously through direct conversion of C-terminal protein thioesters with small-molecule amines^41,72^.

### Enzymatic assembly of linkage-specific ubiquitin chains

K6-linked, K11-linked and K48-linked tetraubiquitin chains and K63-linked diubiquitin, triubiquitin and tetraubiquitin chains were assembled enzymatically from ubiquitin as described previously^66^. Reactions were performed at 37 °C, monitored by intact protein mass spectrometry and stopped with 5 mM DTT. Typical reaction times ranged from 1 to 8 h. For purification, reaction mixtures were diluted in buffer F and subjected to cation-exchange chromatography using a Resource S (ResS) column with an elution gradient from 0 to 1,000 mM NaCl in buffer F and SEC on a HiLoad 16/600 Superdex 75pg column in PBS. Enzymatically assembled K29-linked and K33-linked tetraubiquitin chains were purchased from Bio-Techne (UC-83-025 and UC-103-025).

Enzymatic assembly reactions were also used for the generation of ubiquitin chains containing amino acid changes. Details are provided below.

### Generation of fluorescent TAMRA–maleimide-labeled, K63-linked ubiquitin chains

Ubiquitin–KG^TAMRA^ was obtained as reported previously^72^. Introduction of a C-terminal cysteine and alanine to a shortened ubiquitin (Ub^1–75^-CA) enabled fluorescent labeling of the proximal ubiquitin in polyubiquitin chains by TAMRA–maleimide as follows: K63-linked diubiquitin chains containing Ub^1^^–^^75^-CA as proximal ubiquitin (Ub^K63R^–Ub^1^^–^^75^-CA) were enzymatically assembled with 900 µM Ub^K63R^ and 1.3 mM Ub^1^^–^^75^-CA for 3 h at 37 °C in K63 assembly buffer (40 mM Tris pH 8.5, 10 mM MgCl_2_, 1 µM E1, 8 µM UbEV1, 8 µM UbE2N and 10 mM adenosine triphosohate). K63-linked triubiquitin chains containing Ub^1^^–^^75^-CA as proximal ubiquitin (Ub_2_–Ub^1^^–^^75^-CA) were obtained by using 180 µM K63-linked diubiquitin and 1,800 µM Ub^1^^–^^75^-CA in K63 assembly buffer to favor formation of triubiquitin over tetraubiquitin. Reactions were incubated at 37 °C until diubiquitin was consumed (monitored by protein mass spectrometry). To obtain K63-linked tetraubiquitin chains containing Ub^1^^–^^75^-CA as proximal ubiquitin (Ub_3_–Ub^1^^–^^75^-CA), 80 µM Ub_2_–Ub^1^^–^^75^-CA was combined with 500 µM Ub^K63R^ in K63 assembly buffer and incubated for 2 h at 37 °C.

The resulting substrates were purified on a ResS column with an elution gradient from 0 to 1,000 mM NaCl in buffer F (50 mM sodium acetate pH 4.5). Next, Ub^K63R^–Ub^1^^–^^75^-CA, Ub_2_–Ub^1^^–^^75^-CA and Ub^K63R^–Ub_2_–Ub^1^^–^^75^-CA were buffer-exchanged into labeling buffer (1× PBS and 1 mM TCEP) and fluorescently labeled by the addition of a 1.1 molar equivalent of TAMRA–maleimide. After 1-h incubation at room temperature (RT), the reaction was stopped with 5 mM DTT. Excess dye was removed by dialysis and SEC on a HiLoad 16/600 Superdex 75pg column in PBS. Samples were protected from light from the labeling reaction onward.

### Generation of K63-linked diubiquitin–PA

K63-linked diubiquitin–PA was obtained by mixing 750 µM ubiquitin–PA with 800 µM Ub^K63R^ for 16 h at RT in K63 assembly buffer. Purification on a ResS column with an elution gradient from 0 to 1,000 mM NaCl in buffer F and on a HiLoad 16/600 Superdex 75pg column in buffer A yielded pure K63-linked diubiquitin–PA.

### Crystallization

Crystallization screens were set up by a mosquito HTS robot (TTP Labtech) in 96-well sitting drop vapor diffusion plates of Medical Research Council (MRC) format (Molecular Dimensions) and were incubated at 20 °C. Commercially available plates were used for coarse screening, whereas reservoir solutions of fine-screening plates were prepared with a Dragonfly robot (TTP Labtech). The crystal used for structure determination was grown in a 500 + 500-nl drop prepared from 13 mg ml^−1^ USP54 conjugated to K63-linked diubiquitin–PA in 20 mM Tris pH 8.0, 100 mM NaCl and 4 mM DTT and a reservoir containing 100 mM HEPES pH 6.8, 27.2% (v/v) PEG400 and 200 mM CaCl_2_. Cryoprotection was achieved by soaking in 100 mM HEPES pH 7.3, 30% (v/v) PEG400 and 200 mM CaCl_2_ followed by vitrification in liquid nitrogen.

### Data collection, structure solution and refinement

Diffraction data for crystals of USP54 conjugated to K63-linked diubiquitin–PA were collected at 100 K at the Swiss Light Source (SLS, Paul Scherrer Institute) on beamline PX2. Datasets were integrated using DIALS^73^ and scaled using AIMLESS^74^. Positions and identities of the zinc atoms and all protein chains were obtained from an anomalous dataset (collected at a wavelength of 1.2823 Å) using the CRANK2 pipeline^75^. The structure was solved by molecular replacement from a native dataset (collected at a wavelength of 1.000 Å) using MR Phaser^76^ with a truncated AlphaFold model of USP54 (AF-Q70EL1-F1) and the structure of free ubiquitin (Protein Data Bank (PDB) 1UBQ) as search models, whose relative orientations were obtained from the CRANK2 output. The final model was obtained by multiple cycles of structure building in Coot^77^ and refinement with phenix.refine^78^. Data collection and refinement statistics are shown in Supplementary Table 2. Data were deposited to the PDB under accession code 8C61. Interface characteristics were determined from the Proteins, Interfaces, Structures and Assemblies webserver (https://www.ebi.ac.uk/pdbe/pisa/).

### SEC–MALS

SEC–MALS measurements were performed on an Agilent Infinity II high-performance liquid chromatography (HPLC) system coupled to a Wyatt DAWN NEON 8 angle detector, an Optilab online refractive index detector and an Agilent VWD ultraviolet detector. Protein solutions (60 µl at 2 mg ml^−1^) were injected and separated on a Superdex 75 Increase 10/300 column (Cytiva) with a flow of 0.5 ml min^−1^ in 20 mM Tris pH 8.0 and 100 mM NaCl. Masses were determined with the program Astra (Wyatt Technology) using BSA for calibration.

### Analytical protein labeling

DUBs and probes were mixed in a 1:3 ratio in buffer A (20 mM Tris pH 7.7, 100 mM NaCl and 5 mM DTT) (Extended Data Fig. 2e,f) or buffer B (1× PBS and 5 mM DTT) (Fig. 1d and Extended Data Fig. 1c) to final concentrations of 2–3.5 µM DUB and 6–10.5 µM probe, followed by incubation for 1 h at RT (Extended Data Fig. 2e,f) or 37 °C (Fig. 1d and Extended Data Fig. 1c).

### Ubiquitin–RhoG and Ubl–RhoG cleavage assay

For DUB activity assays using fluorogenic ubiquitin–RhoG and Ubl–RhoG substrates, solutions of 2× DUB (final concentrations: 2 nM–4 µM) with 2× ubiquitin–RhoG and Ubl–RhoG (final concentration: 50 nM) were prepared in 20 mM Tris pH 7.7, 100 mM NaCl, 5 mM DTT and 0.05 mg ml^−1^ BSA. Then, 10 µl of 2× DUB solution were added in triplicate to black 384-well low-volume nonbinding surface plates (Greiner). Reactions were started by the addition of 10 µl of 2× ubiquitin and Ubl RhoG to each well. Substrate cleavage was monitored by fluorescence measurement of liberated RhoG (excitation: 492 nm, emission: 525 nm) on a TECAN Spark plate reader for 1 h at 25–30 °C.

### Fluorescence polarization activity assay

The cleavage of fluorescent ubiquitin chains of different lengths was monitored by fluorescence polarization measurements in black 384-well low-volume nonbinding surface plates (Greiner) on a TECAN Spark plate reader for 2 h at 25 °C (excitation: 535 nm, emission: 595 nm). Substrate and enzyme solutions were prepared separately in FP buffer (20 mM Tris pH 7.7, 100 mM NaCl, 5 mM DTT and 0.1 mg ml^−1^ BSA). Then, 10 µl of 2× substrate solution (final concentration: 50 nM) was added to the plates and preincubated for 10 min. Reactions were started by addition of 10 µl of 2× enzyme solution (final concentrations: 0.0625–4 µM) to each well. Observed rate constants (*k*_obs_) were calculated from averaged technical triplicates using GraphPad Prism and the function ‘plateau followed by one phase decay’. Catalytic efficiencies were obtained as the slope from linear fitting of *k*_obs_ plotted against enzyme concentration.

### Gel-based ubiquitin chain cleavage assay

Ubiquitin chains as 2× substrate dilutions (final concentration: 2–4 µM) and 2× enzyme solutions (final concentration: 0.3–3 µM) were prepared in buffer A (Figs. 2b,d, 5g and 6d,e and Extended Data Fig. 3a,c) or buffer B (Figs. 1f and 2e and Extended Data Figs. 3e,g and 6c) and equilibrated to reaction temperature. Reactions were started by mixing substrate and enzyme solutions in a 1:1 ratio, followed by incubation at 37 °C for most experiments and at RT for experiments shown in Figs. 2e and 5g and Extended Data Fig. 3c,e. Samples were taken at the indicated time points and substrate cleavage was analyzed by SDS–PAGE on NuPAGE Bis–Tris gradient gels (4–12%, Invitrogen) and Coomassie staining. Cleavage of fluorescent TAMRA-labeled ubiquitin chains was additionally visualized by in-gel fluorescence using the Alexa546 channel on a Chemidoc MP Imaging System (Bio-Rad). Protein levels were quantified by densitometry with the program ImageJ. For quantitative evaluation of DUB cleavage activity, the intensities for remaining substrate levels were normalized to the intensities for initial substrate levels. Averaged ratio and s.d. values were calculated from 3–4 independent cleavage assays.

### GFP–ubiquitin cleavage assay

GFP-tagged diubiquitin, triubiquitin and tetraubiquitin substrates with K63 or K48 linkages were assembled from ubiquitinated, LACE-tagged GFP^44^. Purified GFP–ubiquitin substrates (final concentration: 1 µM) and enzyme solutions (final concentration: 0.3 µM for USP54 and 0.5 µM for USP53) were prepared in 20 mM Tris pH 7.7, 100 mM NaCl and 5 mM DTT as 2× dilutions. Solutions were preheated to the reaction temperature of 37 °C and mixed in a 1:1 ratio. Samples were taken at indicated time points and subsequently analyzed by intact protein mass spectrometry or SDS–PAGE. Protein bands on gels were visualized by in-gel fluorescence using the Alexa 488 channel and subsequent Coomassie staining.

### Intact protein mass spectrometry

Protein samples were diluted to ~0.5–1 mg ml^−1^ in PBS and desalted on an AdvanceBio desalting reverse-phase cartridge (Agilent) on an Agilent 1260 II Infinity system before analysis on a mass-selective detection mass analyzer as described previously^41^. Spectral deconvolution was performed with the program ProMass (Enovatia).

### Protein stability measurements

The thermal stability of apo and probe-labeled proteins was determined by a TSA. Samples were diluted in TSA assay buffer (20 mM HEPES pH 7.5, 100 mM NaCl and 5 mM DTT) and probe labeling was conducted with a 3× excess of probe overnight at 4 °C. From these, 2× protein solutions (6 µM) were prepared and mixed with SYPRO Orange dye (6×, in TSA assay buffer) in a 1:1 ratio. USP54^F161K^-containing proteins were purified before analysis and were assayed in a PBS–Tris pH 8.0 buffer mixture. Samples were added to white 96-well PCR plates (Bio-Rad) and a 20–90 °C gradient was applied (increment: 0.3 °C, held for 5 s before read) during which the fluorescence intensity was monitored with a CFX connect real-time PCR system (Bio-Rad) using the fluorescence resonance energy transfer channel (excitation: 450–490 nm, emission: 560–580 nm). Data were analyzed with CFX Maestro.

### Lentivirus generation

Lenti-X 293T cells were transfected using PEI and a 4:3:2 ratio of Tet-pLKO-puro-shRNA:pPAX2:pMD2.G plasmids in Opti-MEM medium and the medium was exchanged on the next day. Viral supernatant was then collected 48 h, 72 h and 96 h after transfection. The pooled supernatant was centrifuged for 5 min and subsequently filtered. The filtered supernatant was concentrated using concentrating filter units (Amicon Ultra-15, 100-kDa cutoff, Sigma-Aldrich) for 30 min to 1 h at 4,000*g* and stored at −80 °C.

### Cell culture, transfection and transduction

HeLa cells were obtained from the Leibniz Institute DSMZ (German Collection of Microorganisms and Cell Cultures). Human embryonic kidney Lenti-X 293T cells were obtained from Takara Bio and CaCo-2 cells were obtained from the American Type Culture Collection. Cells were cultivated in a humidified incubator at 37 °C and 5% CO_2_ in DMEM supplemented with either 10% (HeLa and Lenti-X 293 T) or 20% (CaCo-2) FBS and penicillin–streptomycin. Cells tested negative for *Mycoplasma*. For the creation of doxycycline-inducible knockdown cell lines, the medium was changed to fresh culture medium containing 8 μg ml^−1^ polybrene (Sigma-Aldrich). Concentrated lentivirus was added dropwise. Then, 24 h after infection, virus-containing medium was exchanged to fresh medium. Cell selection was started 48 h after infection and carried out by gradually increasing puromycin (12–16 μg ml^−1^) over the course of 10 days. Knockdown was confirmed by western blot after treatment with 2 μg ml^−1^ doxycycline for 72 h and medium exchange every 24 h. Cells were harvested with RIPA buffer (50 mM Tris HCl pH 8, 150 mM NaCl, 1% IGEPAL CA-630, 0.5% sodium deoxycholate and 0.1% SDS).

### Mass spectrometric analysis of active DUBs

Identification of HA–ubiquitin–PA probe-labeled proteins by mass spectrometry was carried out as described previously^41^. In short, HeLa cell lysate was incubated with HA–ubiquitin–PA or with HA–UFM1–PA as control. Probe-labeled proteins were enriched by HA pulldown and subsequently identified by mass spectrometry.

### Western blotting

Proteins were transferred to a nitrocellulose membrane using a Trans-Blot Turbo system (Bio-Rad) at 1.0 A, 25 V, 30 min for ubiquitin chain analysis or 1.3 A, 25 V, 10 min for all other blots. Membranes were blocked with 5% (w/v) nonfat milk in PBS-T buffer and incubated with indicated primary antibodies (anti-USP53, 1:1,000, Sigma-Aldrich, HPA035844; anti-MARVELD2, 1:500, Thermo Fisher Scientific, 700191; anti-tubulin, 1:4,000, Sigma-Aldrich, T6199; anti-GAPDH, 1:10,000, Thermo Fisher Scientific, AM4300) overnight at 4 °C. Membranes were then incubated with the respective secondary antibody (anti-mouse, 1:5,000, Sigma-Aldrich, NXA931; anti-rabbit, 1:5,000, Sigma-Aldrich, GENA934) coupled to horseradish peroxidase. The chemiluminescent reaction was initiated using a Clarity Western enhanced chemiluminescence substrate and images were taken on a ChemiDoc MP Imaging System (Bio-Rad).

### Ubiquitinome sample preparation

CaCo-2 cells stably transduced with *USP53* shRNA5 were seeded in eight 10-cm dishes, with each dish giving rise to one analyzed sample. Knockdown of *USP53* was induced in four dishes using 2 μg ml^−1^ doxycycline 24 h after seeding and ultrapure water was used as the control in the other four dishes. The medium was changed every 24 h to medium with the respective treatment solution. Then, 72 h after induction of the knockdown, cells were washed thrice in ice-cold PBS and lysed in lysis buffer (50 mM Tris HCl pH 8.5 and 1% (w/v) sodium deoxycholate). Cells were scraped off the dish and proteins were denatured at 95 °C for 10 min. Cell lysates were snap-frozen in liquid nitrogen. Cells were lysed further using sonication in a Bioruptor Pico (Diagenode). Protein concentrations were measured using the BCA assay (Thermo Fisher Scientific). Diglycine-modified peptides were enriched by immunoprecipitation using the PTMScan ubiquitin remnant motif (K-ε-GG) antibody–bead conjugate (kit 5562, Cell Signaling Technology) starting from 800 µg of total protein according to the manufacturer’s protocol. Unbound peptides were removed by washing and the captured peptides were eluted with a low-pH buffer. Eluted peptides were analyzed by nanoflow LC–MS/MS.

### Nanoflow LC–MS/MS

Mass spectrometry was performed on a Vanquish Neo ultra-HPLC System (Thermo Fisher Scientific) coupled to an Exploris 480 Orbitrap mass spectrometer (Thermo Fisher Scientific), operating in positive mode and equipped with a nanospray source. Peptide mixtures were separated on a CSH130 2.5-µm reverse-phase column (Waters; column dimensions 30 cm × 75 µm, packed in-house) using a linear gradient from 0% to 40% B (A = 0.1% formic acid in water; B = 80 % (v/v) acetonitrile in water, with 0.1% formic acid) in 80 min and at a constant flow rate of 300 nl min^−1^. The column eluent was directly sprayed into the electrospray ionization source of the mass spectrometer. All spectra were recorded in DIA mode at a resolution of 120,000 for full scans in the scan range from 350 to 1,650 *m*/*z*. The maximum injection time was set to 50 ms (automatic gain control (AGC) target: 4 × 10^5^). For MS2 acquisition, the mass range was set to 336–1,391 *m*/*z* with dynamic isolation windows ranging from 7 to 82 *m*/*z* with a window overlap of 1 *m*/*z*. The orbitrap resolution for MS2 scans was set to 30,000. The maximum injection time was at 54 ms (AGC target: 5 × 10^4^; normalized AGC target: 100%). A single LC–MS/MS run was performed for all immunoprecipitated peptide material from one sample.

### Ubiquitinome data analysis

DIA raw data files were analyzed with the Spectronaut Pulsar X software package (version 17.0.221202, www.biognosys.com), using directDIA for DIA analysis including MaxLFQ as the label-free quantitation method and Spectronaut’s IDPicker algorithm for protein inference. The *Q*-value cutoff at the precursor and protein level was set to 0.01. All imputation of missing values was disabled. Data were further analyzed in Perseus (version 2.0.7.0). Peptide mass spectral intensity values were log_2_-transformed. Ubiquitinated peptides were filtered for sites that were quantified in all samples and in which the modified lysine was unambiguously identified. Increased ubiquitination sites upon *USP53* knockdown were determined with a significance threshold of −log_10_(*P* value) ≥ 1 and a fold change threshold of log_2_(change in abundance) ≥ 1.

### Preparation of ubiquitin enrichment reagents

OtUBD with an N-terminal cysteine (111 µM) in SEC buffer (20 mM Tris, 100 mM NaCl, 5 mM EDTA and 4 mM TCEP, pH 8.5) was incubated after fresh addition of TCEP (one equivalent) for 30 min. Biotin–PEG2–iodoacetamide (ten equivalents) was then added and the reaction mixture was incubated at 22 °C for 4 h. The protein was purified by a five-step sequential dialysis into storage buffer (20 mM Tris, 100 mM NaCl and 5% glycerol, pH 8). The protein concentration of OtUBD was determined by a DC assay (Bio-Rad). Purified TUBE proteins were biotinylated as described previously^79^. Protein concentrations of TUBE proteins were determined by a microBCA protein assay (Thermo Fisher Scientific). The identity and purity of all biotinylated proteins were checked by SDS–PAGE and intact protein mass spectrometry. Samples were aliquoted and stored at −80 °C.

### Enrichment of endogenously ubiquitinated proteins

CaCo-2 cells (stably transduced with *USP53* shRNA where indicated) were treated as described above for the ubiquitinome analysis. Then, 72 h after induction of the knockdown, cells were washed twice with ice-cold PBS and lysed in urea lysis buffer (8 M urea, 50 mM Tris HCl pH 8, 150 mM NaCl, 1% IGEPAL, 2 mM EDTA, 5% glycerol, 1× EDTA-free protease inhibitor cocktail, 2 µM MG132, 1 mM PMSF, 10 µM PR619, 20 mM *N*-ethylmaleimide and 4 mM 1,10-phenanthroline) and harvested by scraping. Directly after lysis, the obtained suspension was diluted 1:1 for OtUBD and 1:3 for pan-polyubiquitin and K63-linked polyubiquitin enrichment experiments with dilution buffer (urea lysis buffer devoid of urea). Cells were homogenized using sonication for 12 s (3 s on and 3 s off) at 10% amplitude and centrifuged for 10 min at 14,000*g* and 4 °C.

In parallel, 17 nmol of the biotinylated ubiquitin-binding entities were immobilized on 240 µl of high-capacity neutravidin agarose bead slurry (Pierce, Thermo Fisher Scientific) for 1 h at 4 °C with rotation. Excess reagent was washed away with ice-cold PBS. The beads were then equilibrated in diluted lysis buffer before being split up in two tubes.

For each condition, an equal amount of 8–10 mg of protein (as determined per Bradford assay, in 2 ml of diluted lysis buffer, derived from two confluent 10-cm dishes per condition) was added to the preimmobilized pulldown reagents and incubated for 2 h at 4 °C with rotation. Beads were pelleted by centrifugation at 500*g* for 1 min and the supernatant was removed. The resin was washed once with diluted lysis buffer, once with high-salt buffer (50 mM Tris HCl pH 8 and 1 M NaCl) and once with ice-cold PBS. The pulldown reagent and bound proteins were eluted using 2× LDS sample buffer with 25 mM DTT and boiling for 5 min at 95 °C. After pelleting of the beads at 500*g* for 1 min, the supernatant was transferred and analyzed by SDS–PAGE and western blotting as described above.

### UbiCRest assays

Ubiquitinated proteins were isolated as described above using the OtUBD pulldown reagent. After the abovementioned washing steps, beads were additionally washed twice with water. Subsequently, ubiquitinated proteins were eluted from OtUBD using 50 µl of a 100 mM glycine solution at pH 2.5 with an incubation time of 5 min at RT. Beads were pelleted by centrifugation at 500*g* for 1 min. The supernatant was transferred to a new tube and brought immediately to a neutral pH with 5 µl of 1 M Tris at pH 9.0. This step was repeated once; the resulting supernatants were combined and supplemented with final concentrations of 5 mM DTT, 5 mM MgCl_2_ and 100 mM NaCl.

For UbiCRest assays, supernatants were incubated with USP2 (1 µM), OTUB1* (0.2 µM) or AMSH* (0.2 µM) for 1 h at 37 °C. The reactions were stopped by adding 4× LDS sample buffer (supplemented with 50 mM DTT) and visualized by SDS–PAGE and western blotting as described above.

To assess the activity of the catalytic domains of USP53 or USP54 on ubiquitinated proteins, enzymes were added to the supernatants at final concentrations of 0.5, 2 and 5 µM or 0.3, 1 and 3 µM, respectively. Samples were incubated for 1–2 h at 37 °C and analyzed as described above.

### Statistics and reproducibility

Results were consistently observed in at least two, typically three independent experiments. For the statistical testing of quantitative data for the ubiquitin probe pulldown (Fig. 1a) and diglycine proteomics (Fig. 3e), two-sided *t*-tests were used as implemented in Perseus using 250 randomizations, a false discovery rate of 0.05 and an *s*_0_ of 0.1.

### Reporting summary

Further information on research design is available in the Nature Portfolio Reporting Summary linked to this article.

## Online content

Any methods, additional references, Nature Portfolio reporting summaries, source data, extended data, supplementary information, acknowledgements, peer review information; details of author contributions and competing interests; and statements of data and code availability are available at 10.1038/s41589-024-01777-0.

## Supplementary information

Supplementary Information Supplementary Tables 1 and 2 and References.

Reporting Summary

## Source data

Source Data Fig. 1 Numerical source data.

Source Data Fig. 1 Uncropped gels and blots.

Source Data Fig. 2 Numerical source data.

Source Data Fig. 2 Uncropped gels and blots.

Source Data Fig. 3 Numerical source data.

Source Data Fig. 3 Uncropped gels and blots.

Source Data Fig. 4 Numerical source data.

Source Data Fig. 4 Uncropped gels and blots.

Source Data Fig. 5 Uncropped gels and blots.

Source Data Fig. 6 Numerical source data.

Source Data Fig. 6 Uncropped gels and blots.

Source Data Extended Data Fig. 1 Numerical source data.

Source Data Extended Data Fig. 1 Uncropped gels and blots.

Source Data Extended Data Fig. 2 Numerical source data.

Source Data Extended Data Fig. 2 Uncropped gels and blots.

Source Data Extended Data Fig. 3 Numerical source data.

Source Data Extended Data Fig. 3 Uncropped gels and blots.

Source Data Extended Data Fig. 4 Numerical source data.

Source Data Extended Data Fig. 4 Uncropped gels and blots.

Source Data Extended Data Fig. 5 Numerical source data.

Source Data Extended Data Fig. 6 Uncropped gels and blots.

Source Data Extended Data Fig. 9 Numerical source data.

Source Data Extended Data Fig. 9 Uncropped gels and blots.

## Data Availability

Coordinates and structure factors for the crystal structure of USP54 conjugated to K63-linked diubiquitin–PA were deposited to the PDB under accession code 8C61. Mass spectrometry raw data were deposited to the ProteomeXchange Consortium through the PRIDE partner repository under accession codes PXD038455 and PXD054748. Coordinates of other structures were obtained from the PDB under accession codes 1NBF, 1UBQ, 2AYO, 2IBI, 2ZNV, 3WXF, 3WXG, 3ZNZ, 4BOS, 5E6J, 5L8W, 6FGE, 6UPU and 7NPI. Source data are provided with this paper.

## References

[1] Komander, D. & Rape, M. The ubiquitin code. *Annu. Rev. Biochem.* **81**, 203–229 (2012). 22524316 10.1146/annurev-biochem-060310-170328

[2] Dikic, I. & Schulman, B. A.An expanded lexicon for the ubiquitin code. *Nat. Rev. Mol. Cell Biol.* **24**, 273–287 (2023). 36284179 10.1038/s41580-022-00543-1 PMC9595094

[3] Kim, W. et al. Systematic and quantitative assessment of the ubiquitin-modified proteome. *Mol. Cell* **44**, 325–340 (2011). 21906983 10.1016/j.molcel.2011.08.025 PMC3200427

[4] Mukhopadhyay, D. & Riezman, H. Proteasome-independent functions of ubiquitin in endocytosis and signaling. *Science* **315**, 201–205 (2007). 17218518 10.1126/science.1127085

[5] Pruneda, J. N. et al. The molecular basis for ubiquitin and ubiquitin-like specificities in bacterial effector proteases. *Mol. Cell* **63**, 261–276 (2016). 27425412 10.1016/j.molcel.2016.06.015 PMC4961225

[6] Haahr, P. et al. ZUFSP deubiquitylates K63-linked polyubiquitin chains to promote genome stability. *Mol. Cell* **70**, 165–174 (2018). 29576528 10.1016/j.molcel.2018.02.024

[7] Tsuchiya, H. et al. Ub-ProT reveals global length and composition of protein ubiquitylation in cells. *Nat. Commun.* **9**, 524 (2018). 29410401 10.1038/s41467-018-02869-x PMC5802829

[8] Lutz, J., Hollmuller, E., Scheffner, M., Marx, A. & Stengel, F. The length of a ubiquitin chain: a general factor for selective recognition by ubiquitin-binding proteins. *Angew. Chem. Int. Ed. Engl.* **59**, 12371–12375 (2020). 32301549 10.1002/anie.202003058 PMC7384046

[9] Lu, Y., Lee, B. H., King, R. W., Finley, D. & Kirschner, M. W. Substrate degradation by the proteasome: a single-molecule kinetic analysis. *Science* **348**, 1250834 (2015). 25859050 10.1126/science.1250834 PMC4450770

[10] Ikeda, F., Crosetto, N. & Dikic, I. What determines the specificity and outcomes of ubiquitin signaling? *Cell* **143**, 677–681 (2010). 21111228 10.1016/j.cell.2010.10.026

[11] Kwasna, D. et al. Discovery and characterization of ZUFSP/ZUP1, a distinct deubiquitinase class important for genome stability. *Mol. Cell* **70**, 150–164 (2018). 29576527 10.1016/j.molcel.2018.02.023 PMC5896202

[12] Waltho, A. et al. K48- and K63-linked ubiquitin chain interactome reveals branch- and length-specific ubiquitin interactors. *Life Sci. Alliance* **7**, e202402740 (2024). 38803224 10.26508/lsa.202402740 PMC11109483

[13] Swatek, K. N. & Komander, D. Ubiquitin modifications. *Cell Res.* **26**, 399–422 (2016). 27012465 10.1038/cr.2016.39 PMC4822133

[14] Abdul Rehman, S. A. et al. Mechanism of activation and regulation of deubiquitinase activity in MINDY1 and MINDY2. *Mol. Cell* **81**, 4176–4190 (2021). 34529927 10.1016/j.molcel.2021.08.024 PMC8550791

[15] Mevissen, T. E. et al. OTU deubiquitinases reveal mechanisms of linkage specificity and enable ubiquitin chain restriction analysis. *Cell* **154**, 169–184 (2013). 23827681 10.1016/j.cell.2013.05.046 PMC3705208

[16] Bekes, M. et al. Recognition of Lys48-linked di-ubiquitin and deubiquitinating activities of the SARS coronavirus papain-like protease. *Mol. Cell* **62**, 572–585 (2016). 27203180 10.1016/j.molcel.2016.04.016 PMC4875570

[17] Shin, D. et al. Papain-like protease regulates SARS-CoV-2 viral spread and innate immunity. *Nature* **587**, 657–662 (2020). 32726803 10.1038/s41586-020-2601-5 PMC7116779

[18] Beck, D. B. et al. Linkage-specific deubiquitylation by OTUD5 defines an embryonic pathway intolerant to genomic variation. *Sci. Adv.* **7**, eabe2116 (2021). 33523931 10.1126/sciadv.abe2116 PMC7817106

[19] Damgaard, R. B. et al. The deubiquitinase OTULIN is an essential negative regulator of inflammation and autoimmunity. *Cell* **166**, 1215–1230 (2016). 27523608 10.1016/j.cell.2016.07.019 PMC5002269

[20] Clague, M. J., Urbe, S. & Komander, D. Breaking the chains: deubiquitylating enzyme specificity begets function. *Nat. Rev. Mol. Cell Biol.* **20**, 338–352 (2019). 30733604 10.1038/s41580-019-0099-1

[21] Tsou, W. L. et al. Systematic analysis of the physiological importance of deubiquitinating enzymes. *PLoS ONE* **7**, e43112 (2012). 22937016 10.1371/journal.pone.0043112 PMC3427330

[22] Abdul Rehman, S. A. et al. MINDY-1 is a member of an evolutionarily conserved and structurally distinct new family of deubiquitinating enzymes. *Mol. Cell* **63**, 146–155 (2016). 27292798 10.1016/j.molcel.2016.05.009 PMC4942677

[23] Hewings, D. S. et al. Reactive-site-centric chemoproteomics identifies a distinct class of deubiquitinase enzymes. *Nat. Commun.* **9**, 1162 (2018). 29563501 10.1038/s41467-018-03511-6 PMC5862848

[24] Faesen, A. C. et al. The differential modulation of USP activity by internal regulatory domains, interactors and eight ubiquitin chain types. *Chem. Biol.* **18**, 1550–1561 (2011). 22195557 10.1016/j.chembiol.2011.10.017

[25] Hermanns, T. et al. A family of unconventional deubiquitinases with modular chain specificity determinants. *Nat. Commun.* **9**, 799 (2018). 29476094 10.1038/s41467-018-03148-5 PMC5824887

[26] Sato, Y. et al. Structural basis for specific cleavage of Lys 63-linked polyubiquitin chains. *Nature* **455**, 358–362 (2008). 18758443 10.1038/nature07254

[27] Walden, M. et al. Metabolic control of BRISC–SHMT2 assembly regulates immune signalling. *Nature* **570**, 194–199 (2019). 31142841 10.1038/s41586-019-1232-1 PMC6914362

[28] Rabl, J. et al. Structural basis of BRCC36 function in DNA repair and immune regulation. *Mol. Cell* **75**, 483–497 (2019). 31253574 10.1016/j.molcel.2019.06.002 PMC6695476

[29] Mevissen, T. E. T. & Komander, D. Mechanisms of deubiquitinase specificity and regulation. *Annu. Rev. Biochem.* **86**, 159–192 (2017). 28498721 10.1146/annurev-biochem-061516-044916

[30] Kazmierczak, M. et al. Progressive hearing loss in mice carrying a mutation in *Usp53*. *J. Neurosci.* **35**, 15582–15598 (2015). 26609154 10.1523/JNEUROSCI.1965-15.2015 PMC4659823

[31] Fraile, J. M., Campos-Iglesias, D., Rodriguez, F., Espanol, Y. & Freije, J. M. The deubiquitinase USP54 is overexpressed in colorectal cancer stem cells and promotes intestinal tumorigenesis. *Oncotarget* **7**, 74427–74434 (2016). 27769071 10.18632/oncotarget.12769 PMC5342676

[32] Ye, Y., Scheel, H., Hofmann, K. & Komander, D. Dissection of USP catalytic domains reveals five common insertion points. *Mol. Biosyst.* **5**, 1797–1808 (2009). 19734957 10.1039/b907669g

[33] Quesada, V. et al. Cloning and enzymatic analysis of 22 novel human ubiquitin-specific proteases. *Biochem. Biophys. Res. Commun.* **314**, 54–62 (2004). 14715245 10.1016/j.bbrc.2003.12.050

[34] Alhebbi, H. et al. New paradigms of *USP53* disease: normal GGT cholestasis, BRIC, cholangiopathy, and responsiveness to rifampicin. *J. Hum. Genet* **66**, 151–159 (2021). 32759993 10.1038/s10038-020-0811-1

[35] Bull, L. N. et al. Cholestasis due to USP53 deficiency. *J. Pediatr. Gastroenterol. Nutr.* **72**, 667–673 (2021). 33075013 10.1097/MPG.0000000000002926 PMC8549450

[36] Maddirevula, S. et al. Identification of novel loci for pediatric cholestatic liver disease defined by KIF12, PPM1F, USP53, LSR, and WDR83OS pathogenic variants. *Genet. Med.* **21**, 1164–1172 (2019). 30250217 10.1038/s41436-018-0288-x

[37] Zhang, J. et al. Low-GGT intrahepatic cholestasis associated with biallelic *USP53* variants: clinical, histological and ultrastructural characterization. *Liver Int.* **40**, 1142–1150 (2020). 32124521 10.1111/liv.14422

[38] Pinto-Fernandez, A. et al. Comprehensive landscape of active deubiquitinating enzymes profiled by advanced chemoproteomics. *Front. Chem.* **7**, 592 (2019). 31555637 10.3389/fchem.2019.00592 PMC6727631

[39] Borodovsky, A. et al. A novel active site-directed probe specific for deubiquitylating enzymes reveals proteasome association of USP14. *EMBO J.* **20**, 5187–5196 (2001). 11566882 10.1093/emboj/20.18.5187 PMC125629

[40] Ekkebus, R. et al. On terminal alkynes that can react with active-site cysteine nucleophiles in proteases. *J. Am. Chem. Soc.* **135**, 2867–2870 (2013). 23387960 10.1021/ja309802n PMC3585465

[41] O’Dea, R. et al. Molecular basis for ubiquitin/Fubi cross-reactivity in USP16 and USP36. *Nat. Chem. Biol.* **19**, 1394–1405 (2023). 37443395 10.1038/s41589-023-01388-1 PMC10611586

[42] Sato, Y. et al. Structures of CYLD USP with Met1- or Lys63-linked diubiquitin reveal mechanisms for dual specificity. *Nat. Struct. Mol. Biol.* **22**, 222–229 (2015). 25686088 10.1038/nsmb.2970

[43] Ritorto, M. S. et al. Screening of DUB activity and specificity by MALDI-TOF mass spectrometry. *Nat. Commun.* **5**, 4763 (2014). 25159004 10.1038/ncomms5763 PMC4147353

[44] Hofmann, R., Akimoto, G., Wucherpfennig, T. G., Zeymer, C. & Bode, J. W. Lysine acylation using conjugating enzymes for site-specific modification and ubiquitination of recombinant proteins. *Nat. Chem.* **12**, 1008–1015 (2020). 32929246 10.1038/s41557-020-0528-y

[45] Bekes, M. et al. SARS hCoV papain-like protease is a unique Lys48 linkage-specific di-distributive deubiquitinating enzyme. *Biochem. J.* **468**, 215–226 (2015). 25764917 10.1042/BJ20141170 PMC4447217

[46] Lee, B. H. et al. USP14 deubiquitinates proteasome-bound substrates that are ubiquitinated at multiple sites. *Nature* **532**, 398–401 (2016). 27074503 10.1038/nature17433 PMC4844788

[47] Riazuddin, S. et al. Tricellulin is a tight-junction protein necessary for hearing. *Am. J. Hum. Genet* **79**, 1040–1051 (2006). 17186462 10.1086/510022 PMC1698716

[48] Ikenouchi, J. et al. Tricellulin constitutes a novel barrier at tricellular contacts of epithelial cells. *J. Cell Biol.* **171**, 939–945 (2005). 16365161 10.1083/jcb.200510043 PMC2171318

[49] Masuda, S. et al. LSR defines cell corners for tricellular tight junction formation in epithelial cells. *J. Cell Sci.* **124**, 548–555 (2011). 21245199 10.1242/jcs.072058

[50] Kamitani, T. et al. Deletion of tricellulin causes progressive hearing loss associated with degeneration of cochlear hair cells. *Sci. Rep.* **5**, 18402 (2015). 26677943 10.1038/srep18402 PMC4683410

[51] Higashi, T. et al. Analysis of the ‘angulin’ proteins LSR, ILDR1 and ILDR2—tricellulin recruitment, epithelial barrier function and implication in deafness pathogenesis. *J. Cell Sci.* **126**, 966–977 (2013). 23239027 10.1242/jcs.116442

[52] Zhang, M., Berk, J. M., Mehrtash, A. B., Kanyo, J. & Hochstrasser, M. A versatile new tool derived from a bacterial deubiquitylase to detect and purify ubiquitylated substrates and their interacting proteins. *PLoS Biol.* **20**, e3001501 (2022). 35771886 10.1371/journal.pbio.3001501 PMC9278747

[53] Berk, J. M. et al. A deubiquitylase with an unusually high-affinity ubiquitin-binding domain from the scrub typhus pathogen *Orientia tsutsugamushi*. *Nat. Commun.* **11**, 2343 (2020). 32393759 10.1038/s41467-020-15985-4 PMC7214410

[54] Hjerpe, R. et al. Efficient protection and isolation of ubiquitylated proteins using tandem ubiquitin-binding entities. *EMBO Rep.* **10**, 1250–1258 (2009). 19798103 10.1038/embor.2009.192 PMC2775171

[55] Sims, J. J. et al. Polyubiquitin-sensor proteins reveal localization and linkage-type dependence of cellular ubiquitin signaling. *Nat. Methods* **9**, 303–309 (2012). 22306808 10.1038/nmeth.1888 PMC3438894

[56] Flierman, D. et al. Non-hydrolyzable diubiquitin probes reveal linkage-specific reactivity of deubiquitylating enzymes mediated by S2 pockets. *Cell Chem. Biol.* **23**, 472–482 (2016). 27066941 10.1016/j.chembiol.2016.03.009 PMC4850247

[57] Samara, N. L. et al. Structural insights into the assembly and function of the SAGA deubiquitinating module. *Science* **328**, 1025–1029 (2010). 20395473 10.1126/science.1190049 PMC4220450

[58] Kohler, A., Zimmerman, E., Schneider, M., Hurt, E. & Zheng, N. Structural basis for assembly and activation of the heterotetrameric SAGA histone H2B deubiquitinase module. *Cell* **141**, 606–617 (2010). 20434206 10.1016/j.cell.2010.04.026 PMC2901531

[59] Hu, M. et al. Crystal structure of a UBP-family deubiquitinating enzyme in isolation and in complex with ubiquitin aldehyde. *Cell* **111**, 1041–1054 (2002). 12507430 10.1016/s0092-8674(02)01199-6

[60] Klemm, T. et al. Mechanism and inhibition of the papain-like protease, PLpro, of SARS-CoV-2. *EMBO J.* **39**, e106275 (2020). 32845033 10.15252/embj.2020106275 PMC7461020

[61] Jonsson, E., Htet, Z. M., Bard, J. A. M., Dong, K. C. & Martin, A. Ubiquitin modulates 26S proteasome conformational dynamics and promotes substrate degradation. *Sci. Adv.* **8**, eadd9520 (2022). 36563145 10.1126/sciadv.add9520 PMC9788759

[62] Reyes-Turcu, F. E. et al. The ubiquitin binding domain ZnF UBP recognizes the C-terminal diglycine motif of unanchored ubiquitin. *Cell* **124**, 1197–1208 (2006). 16564012 10.1016/j.cell.2006.02.038

[63] Amendola, M. & Squires, J.E. Pediatric genetic cholestatic liver disease overview. In *Gene Reviews* (eds Adam, M.P. et al.) (University of Washington, 1993). 36108118

[64] Sambrotta, M. et al. Mutations in *TJP2* cause progressive cholestatic liver disease. *Nat. Genet.* **46**, 326–328 (2014). 24614073 10.1038/ng.2918 PMC4061468

[65] Swatek, K. N. et al. Insights into ubiquitin chain architecture using Ub-clipping. *Nature* **572**, 533–537 (2019). 31413367 10.1038/s41586-019-1482-y PMC6823057

[66] Michel, M. A., Komander, D. & Elliott, P. R. Enzymatic assembly of ubiquitin chains. *Methods Mol. Biol.* **1844**, 73–84 (2018). 30242704 10.1007/978-1-4939-8706-1_6

[67] Gersch, M. et al. Distinct USP25 and USP28 oligomerization states regulate deubiquitinating activity. *Mol. Cell* **74**, 436–451 (2019). 30926242 10.1016/j.molcel.2019.02.030 PMC6509359

[68] Bremm, A., Freund, S. M. & Komander, D. Lys11-linked ubiquitin chains adopt compact conformations and are preferentially hydrolyzed by the deubiquitinase Cezanne. *Nat. Struct. Mol. Biol.* **17**, 939–947 (2010). 20622874 10.1038/nsmb.1873 PMC2917782

[69] Hospenthal, M. K., Freund, S. M. & Komander, D. Assembly, analysis and architecture of atypical ubiquitin chains. *Nat. Struct. Mol. Biol.* **20**, 555–565 (2013). 23563141 10.1038/nsmb.2547 PMC4176834

[70] Michel, M. A. et al. Assembly and specific recognition of K29- and K33-linked polyubiquitin. *Mol. Cell* **58**, 95–109 (2015). 25752577 10.1016/j.molcel.2015.01.042 PMC4386031

[71] Pickart, C. M. & Raasi, S. Controlled synthesis of polyubiquitin chains. *Methods Enzymol.* **399**, 21–36 (2005). 16338346 10.1016/S0076-6879(05)99002-2

[72] Zhao, Z., O’Dea, R., Wendrich, K., Kazi, N. & Gersch, M. Native semisynthesis of isopeptide-linked substrates for specificity analysis of deubiquitinases and Ubl proteases. *J. Am. Chem. Soc.* **145**, 20801–20812 (2023). 37712884 10.1021/jacs.3c04062 PMC10540217

[73] Beilsten-Edmands, J. et al. Scaling diffraction data in the DIALS software package: algorithms and new approaches for multi-crystal scaling. *Acta Crystallogr. D Struct. Biol.* **76**, 385–399 (2020). 32254063 10.1107/S2059798320003198 PMC7137103

[74] Evans, P. R. & Murshudov, G. N. How good are my data and what is the resolution? *Acta Crystallogr. D Biol. Crystallogr.* **69**, 1204–1214 (2013). 23793146 10.1107/S0907444913000061 PMC3689523

[75] Skubak, P. et al. A new MR-SAD algorithm for the automatic building of protein models from low-resolution X-ray data and a poor starting model. *IUCrJ* **5**, 166–171 (2018). 29765606 10.1107/S2052252517017961 PMC5947721

[76] McCoy, A. J. et al. Phaser crystallographic software. *J. Appl. Crystallogr.* **40**, 658–674 (2007). 19461840 10.1107/S0021889807021206 PMC2483472

[77] Emsley, P., Lohkamp, B., Scott, W. G. & Cowtan, K. Features and development of Coot. *Acta Crystallogr. D Struct. Biol.* **66**, 486–501 (2010). 10.1107/S0907444910007493 PMC2852313 20383002

[78] Adams, P. D. et al. The PHENIX software for automated determination of macromolecular structures. *Methods* **55**, 94–106 (2011). 21821126 10.1016/j.ymeth.2011.07.005 PMC3193589

[79] Fairhead, M. & Howarth, M. Site-specific biotinylation of purified proteins using BirA. *Methods Mol. Biol.* **1266**, 171–184 (2015). 25560075 10.1007/978-1-4939-2272-7_12 PMC4304673

