# Supplementary material for: Discovery and mechanism of K63-linkage-directed deubiquitinase activity in USP53
Source: Nat Chem Biol. 2024 Nov 25;21(5):746–57. doi: 10.1038/s41589-024-01777-0 (PMC12037411; doi:10.1038/s41589-024-01777-0)
Supplement: Supplementary file 1 — Supplementary Tables 1 and 2 and References. [file 41589_2024_1777_MOESM1_ESM.pdf]

nature chemical biology

Supplementary information

https://doi.org/10.1038/s41589-024-01777-0

Discovery and mechanism of K63-linkage-directed deubiquitinase activity in USP53

In the format provided by the authors and unedited

Supplementary Information

Discovery and mechanism of K63-linkage-directed deubiquitinase activity in USP53

Kim Wendrich[1,2,5], Kai Gallant[1,2,5], Sarah Recknagel[1,2], Stavroula Petroulia[1,2], Nafizul Haque Kazi[1,2], Jan André Hane[1,2], Siska Führer[1,2], Karel Bezstarosti[3], Rachel O'Dea[1,2,4], Jeroen Demmers[3] and Malte Gersch[1,2,*]

[1]Max Planck Institute of Molecular Physiology, Chemical Genomics Centre, Dortmund, Germany.

[2]TU Dortmund University, Department of Chemistry and Chemical Biology, Dortmund, Germany.

[3]Erasmus University Medical Center, Proteomics Center, Rotterdam, the Netherlands.

[4]Present address: Medical Research Council Protein Phosphorylation and Ubiquitylation Unit, University of Dundee, Dundee, UK.

[5]These authors contributed equally: Kim Wendrich, Kai Gallant.

*Correspondence: malte.gersch@mpi-dortmund.mpg.de

Contents:

- Supplementary Tables 1 and 2
- Supplementary References

Supplementary Table 1. Patient mutations in *USP53* associated with progressive familial intrahepatic cholestasis.

Mutation (Genetic)	Mutation (Protein)	Reference	Comment
**Missense**			
c.91G > A	p.Gly31Ser	Zheng *et al.*, 2024 [1]	Near active site
c.145G>T	p.Val49Phe	Samanta *et al.*, 2024 [2]	Near active site
c.297G>T	p.Arg99Ser	Zhang *et al.*, 2020 [3]	Near active site
c.395A>G	p.His132Arg	Zhang *et al.*, 2020 [3]	Zn^{2+} coordinating
c.394C > T	p.His132Tyr	Zheng *et al.*, 2024 [1]	Zn^{2+} coordinating
c.682T>C	p.Cys228Arg	Samanta *et al.*, 2024 [2]	Zn^{2+} coordinating
c.725C>T	p.Pro242Leu	Bull *et al.*, 2021 [4]	Within domain
c.878G>T	p.Gly293Val	Zhang *et al.*, 2020 [3]	Within domain
c.908G>A	p.Cys303Tyr	Samanta *et al.*, 2024 [2]	Near active site
c.2869C>T	p.His957Tyr	Samanta *et al.*, 2024 [2]	Outside cat. domain
**Nonsense**			
c.169C>T	p.Arg57*	Zhang *et al.*, 2020 [3]<br>Cheema *et al.*, 2020 [5]	
c.1012C>T	p.Arg338*	Zhang *et al.*, 2020 [3]	
c.1426C>T	p.Arg476*	Zhang *et al.*, 2020 [3]	
c.1558C>T	p.Arg520*	Zhang *et al.*, 2020 [3]<br>Ates *et al.*, 2023 [6]	
c.1744C>T	p.Arg582*	Alhebbi *et al.*, 2021 [7]	
c.145-11_167del	p:?	Bull *et al.*, 2021 [4]	Intron/Exon boundary
Deletion of exon 1	p:?	Bull *et al.*, 2021 [4]	+*MYOZ2* deletion
Deletion of exon 14/15	p:?	Cheema *et al.*, 2023 [8]	
**Frameshift**			
c.475_476delCT	p.Leu159fs	Cheema *et al.*, 2020 [5]	
c.510delA	p.Ser171Argfs*62	Bull *et al.*, 2021 [4]	
c.581delA	p.Arg195Glufs*38	Zhang *et al.*, 2020 [3]	
c.774_774delG	p.Thr259Profs*8	Cheema *et al.*, 2023 [8]	
c.831_832insAG	p:Val279Glufs*16	Zhang *et al.*, 2020 [3]	
c.951delT	p.Phe317Leufs*6	Maddirevula *et al.*, 2019 [9]<br>Cheema *et al.*, 2020 [5]<br>Alhebbi *et al.*, 2021 [7]	
c.1017_1057del	p.Cys339Trpfs*7	Shatokina *et al.*, 2021 [10]	
c.1214dupA	p.Asn405fs	Cheema *et al.*, 2020 [5]	
c.1687_1688delinsC	p.Ser563Profs*25	Porta *et al.*, 2021 [11]	
**Splice site**			
c.238-1G>C	p:?	Gezdirici *et al.*, 2023 [12]	
c.569+2T>C	p:?	Zhang *et al.*, 2020 [3]	
c.822+1delG	p:?	Cheema *et al.*, 2020 [5]<br>Vij *et al.*, 2022 [13]	
c.972+3_972+6del,<br>c.973-1G>A	p:?<br>p:?	Ahn *et al.*, 2023 [14]	

Supplementary Table 2. Data collection and refinement statistics.

	USP54~diUb(K63)-PA (SAD data)	USP54~diUb(K63)-PA (native data, PDB code: 8C61)
**Data collection**		
Beamline	SLS – PX2	SLS – PX2
Wavelength (Å)	1.282	1.000
Space group	$P\ 2_12_12_1$	$P\ 2_12_12_1$
Cell dimensions		
a, *b*, *c* (Å)	122.58, 126.59, 144.22	122.74, 126.56, 144.17
α, β, γ (°)	90, 90, 90	90, 90, 90
Observed reflections	1,859,182 (122,127)	699,375 (71,819)
Unique reflections	69,709 (4,449)	78,247 (7,759)
Resolution (Å)	88.06 – 2.6 (2.66 – 2.6)	63.28 – 2.5 (2.59 – 2.5)
R_{merge}	0.091 (2.410)	0.062 (1.239)
R_{meas}	0.094 (2.498)	0.066 (1.313)
$I/\sigma(I)$	24.5 (2.2)	16.2 (1.7)
$CC_{1/2}$	1.000 (0.789)	0.998 (0.682)
Completeness (%)	100 (100)	100 (100)
Redundancy	26.7 (27.5)	6.6 (6.3)
Wilson *B* (Å^2)	74.4	74.8
**Phasing**		
Method	SAD	MR
Resolution (Å)	2.6	
Anom. completeness (%)	100 (100)	
Anom. multiplicity	13.8 (13.9)	
<FOM>	0.5179	
**Refinement**		
Copies / a.s.u.		4
Resolution (Å)		2.5 Å
No. reflections		78,226
R_{work} / R_{free} (%)		20.0 / 24.3
No. atoms		14,892
Protein		14,647
Ligand		32
Water		213
B factors (Å^2)		95.9
Protein (Å^2)		96.2
Ligand (Å^2)		88.8
Water (Å^2)		79.5
R.m.s.d.		
Bond lengths (Å)		0.004
Bond angles (°)		0.62
Ramachandran (favored / allowed / outlier) (%)		96.8 / 3.2 / 0
Clashscore		8.5
Rotamer outliers (%)		2.8

The dataset was collected from a single crystal. Values in parentheses are for highest-resolution shell. a.s.u., asymmetric unit. R.m.s.d., root mean square deviations.

Supplementary References

1. Zheng, Y., Guo, H., Chen, L., Cheng, W., Yan, K., Zhang, Z., Li, M., Jin, Y., Hu, G., Wang, C., Zhou, C., Zhou, W., Jia, Z., Zheng, B. & Liu, Z. Diagnostic yield and novel candidate genes by next generation sequencing in 166 children with intrahepatic cholestasis. *Hepatol Int* **18**, 661-672 (2024).

2. Samanta, A., Parveen, N., Sen Sarma, M., Poddar, U. & Srivastava, A. Cholestatic Liver Disease due to Novel USP53 Mutations: A Case Series of Three Indian Children. *J Clin Exp Hepatol* **14**, 101290 (2024).

3. Zhang, J., Yang, Y., Gong, J.Y., Li, L.T., Li, J.Q., Zhang, M.H., Lu, Y., Xie, X.B., Hong, Y.R., Yu, Z., Knisely, A.S. & Wang, J.S. Low-GGT intrahepatic cholestasis associated with biallelic USP53 variants: Clinical, histological and ultrastructural characterization. *Liver Int* **40**, 1142-1150 (2020).

4. Bull, L.N., Ellmers, R., Foskett, P., Strautnieks, S., Sambrotta, M., Czubkowski, P., Jankowska, I., Wagner, B., Deheragoda, M. & Thompson, R.J. Cholestasis Due to USP53 Deficiency. *J Pediatr Gastroenterol Nutr* **72**, 667-673 (2021).

5. Cheema, H., Bertoli-Avella, A.M., Skrahina, V., Anjum, M.N., Waheed, N., Saeed, A., Beetz, C., Perez-Lopez, J., Rocha, M.E., Alawbathani, S., Pereira, C., Hovakimyan, M., Patric, I.R.P., Paknia, O., Ameziane, N., Cozma, C., Bauer, P. & Rolfs, A. Genomic testing in 1019 individuals from 349 Pakistani families results in high diagnostic yield and clinical utility. *NPJ Genom Med* **5**, 44 (2020).

6. Ates, B.B., Ceylan, A.C., Hizal, G., Duran, F., Dogan, H.T. & Hizli, S. A novel homozygous mutation in the USP53 gene as the cause of benign recurrent intrahepatic cholestasis in children: a case report. *Turk J Pediatr* **65**, 1012-1017 (2023).

7. Alhebbi, H., Peer-Zada, A.A., Al-Hussaini, A.A., Algubaisi, S., Albassami, A., AlMasri, N., Alrusayni, Y., Alruzug, I.M., Alharby, E., Samman, M.A., Ayoub, S.Z., Maddirevula, S., Peake, R.W.A., Alkuraya, F.S., Wali, S. & Almontashiri, N.A.M. New paradigms of USP53 disease: normal GGT cholestasis, BRIC, cholangiopathy, and responsiveness to rifampicin. *J Hum Genet* **66**, 151-159 (2021).

8. Cheema, H.A., Waheed, N., Saeed, A., Anjum, M.N., Fayyaz, Z. & Ijaz, S. The Mutational Landscape Of Genetic Cholestatic Diseases In Pakistani Children. *J Pak Med Assoc* **73**, 1610-1621 (2023).

9. Maddirevula, S., Alhebbi, H., Alqahtani, A., Algoufi, T., Alsaif, H.S., Ibrahim, N., Abdulwahab, F., Barr, M., Alzaidan, H., Almehaideb, A., AlSasi, O., Alhashem, A., Hussaini, H.A., Wali, S. & Alkuraya, F.S. Identification of novel loci for pediatric cholestatic liver disease defined by KIF12, PPM1F, USP53, LSR, and WDR83OS pathogenic variants. *Genet Med* **21**, 1164-1172 (2019).

10. Shatokhina, O., Semenova, N., Demina, N., Dadali, E., Polyakov, A. & Ryzhkova, O. A Two-Year Clinical Description of a Patient with a Rare Type of Low-GGT Cholestasis Caused by a Novel Variant of USP53. *Genes (Basel)* **12**, 1618 (2021).

11. Porta, G., Rigo, P.S.M., Porta, A., Pugliese, R.P.S., Danesi, V.L.B., Oliveira, E., Borges, C.C.V., Ribeiro, C. & Miura, I.K. Progressive Familial Intrahepatic Cholestasis Associated With USP53 Gene Mutation in a Brazilian Child. *J Pediatr Gastroenterol Nutr* **72**, 674-676 (2021).

12. Gezdirici, A., Kalaycik Sengul, O., Dogan, M., Ozguven, B.Y. & Akbulut, E. Biallelic Novel USP53 Splicing Variant Disrupting the Gene Function that Causes Cholestasis Phenotype and Review of the Literature. *Mol Syndromol* **13**, 471-484 (2023).

13. Vij, M. & Sankaranarayanan, S. Biallelic Mutations in Ubiquitin-Specific Peptidase 53 (USP53) Causing Progressive Intrahepatic Cholestasis. Report of a Case With Review of Literature. *Pediatr Dev Pathol* **25**, 207-212 (2022).

14. Ahn, S., Choi, J. & Jeong, S.H. The First Korean Adult Case of Progressive Familial Intrahepatic Cholestasis Type 7 with Novel USP53 Splicing Variants by Next Generation Sequencing. *Yonsei Med J* **64**, 745-749 (2023).
