# Supplementary material for: Discovery and mechanism of K63-linkage-directed deubiquitinase activity in USP53
Source: Nat Chem Biol. 2024 Nov 25;21(5):746–57. doi: 10.1038/s41589-024-01777-0 (PMC12037411; doi:10.1038/s41589-024-01777-0)
Supplement: Supplementary file 2 — Reporting Summary [file 41589_2024_1777_MOESM2_ESM.pdf]

nature portfolio

Corresponding author(s): Malte Gersch

Last updated by author(s): Oct 6, 2024

Reporting Summary

Nature Portfolio wishes to improve the reproducibility of the work that we publish. This form provides structure for consistency and transparency in reporting. For further information on Nature Portfolio policies, see our Editorial Policies and the Editorial Policy Checklist.

Statistics

For all statistical analyses, confirm that the following items are present in the figure legend, table legend, main text, or Methods section.

n/a	Confirmed	
☐	☒	The exact sample size (*n*) for each experimental group/condition, given as a discrete number and unit of measurement
☒	☐	A statement on whether measurements were taken from distinct samples or whether the same sample was measured repeatedly
☐	☒	The statistical test(s) used AND whether they are one- or two-sided<br>*Only common tests should be described solely by name; describe more complex techniques in the Methods section.*
☒	☐	A description of all covariates tested
☒	☐	A description of any assumptions or corrections, such as tests of normality and adjustment for multiple comparisons
☐	☒	A full description of the statistical parameters including central tendency (e.g. means) or other basic estimates (e.g. regression coefficient) AND variation (e.g. standard deviation) or associated estimates of uncertainty (e.g. confidence intervals)
☐	☒	For null hypothesis testing, the test statistic (e.g. *F*, *t*, *r*) with confidence intervals, effect sizes, degrees of freedom and *P* value noted<br>*Give P values as exact values whenever suitable.*
☒	☐	For Bayesian analysis, information on the choice of priors and Markov chain Monte Carlo settings
☒	☐	For hierarchical and complex designs, identification of the appropriate level for tests and full reporting of outcomes
☒	☐	Estimates of effect sizes (e.g. Cohen's *d*, Pearson's *r*), indicating how they were calculated

Our web collection on statistics for biologists contains articles on many of the points above.

Software and code

Policy information about availability of computer code

Data collection	SparkControl (Tecan, V2.3); CFX Maestro (Bio-Rad, V4.1.2433.1219); ASTRA (Wyatt Technology, V7.3.2.21); OpenLab CDS (Agilent, V2.4)
Data analysis	Image Lab (Bio-Rad, V2.4.0.03); Prism (GraphPad, V9); Pymol (Schrödinger, V2.5.5); ImageJ (W. Rasband/NIH, V1.53); SwissModel Homology Server (V4.0); Promass (Enovatia, V3.0rev12); Dials (V3.5.0); Aimless (V1.12.10); CRANK2 (V2.9.281); MR Phaser (V2.8.3); Coot (V0.7.1); Phenix.Refine (V1.19.2); Spectronaut (V17.0.221202); Perseus (V2.0.7.0)

For manuscripts utilizing custom algorithms or software that are central to the research but not yet described in published literature, software must be made available to editors and reviewers. We strongly encourage code deposition in a community repository (e.g. GitHub). See the Nature Portfolio guidelines for submitting code & software for further information.

Data

Policy information about availability of data

All manuscripts must include a data availability statement. This statement should provide the following information, where applicable:

- Accession codes, unique identifiers, or web links for publicly available datasets
- A description of any restrictions on data availability
- For clinical datasets or third party data, please ensure that the statement adheres to our policy

Coordinates and structure factors for the USP54~diUb(K63)-PA crystal structure have been deposited with the Protein Data Bank (PDB) under accession code 8C61. Mass spectrometry raw data have been deposited with the ProteomeXchange Consortium via the PRIDE partner repository under accession codes PXD038455 and

PXD054748. Coordinates of other structures were obtained from the PDB via accession codes 1NBF, 1UBQ, 2AYO, 2IBI, 2ZNV, 3WXF, 3WXG, 3ZNZ, 4BOS, 5E6J, 5L8W, 6FGE, 6UPU, 7NPI. Source data are provided with this paper, which include mass spectrometry data for Fig. 1a and Fig. 3e as well as uncropped images of all gels and blots.

Human research participants

Policy information about studies involving human research participants and Sex and Gender in Research.

Reporting on sex and gender	Human research participants were not involved in this study.
Population characteristics	Human research participants were not involved in this study.
Recruitment	Human research participants were not involved in this study.
Ethics oversight	Human research participants were not involved in this study.

Note that full information on the approval of the study protocol must also be provided in the manuscript.

Field-specific reporting

Please select the one below that is the best fit for your research. If you are not sure, read the appropriate sections before making your selection.

☒ Life sciences ☐ Behavioural & social sciences ☐ Ecological, evolutionary & environmental sciences

For a reference copy of the document with all sections, see nature.com/documents/nr-reporting-summary-flat.pdf

Life sciences study design

All studies must disclose on these points even when the disclosure is negative.

Sample size	For quantitative experiments, a sample size of 3 to 6 independent experiments was chosen in line with what is the standard of the field in the molecular biosciences. Statistically significant and conclusive results were obtained with the chosen sample sizes.
Data exclusions	Data exclusion occurred in the processing of crystallographic data as implemented in the respective software (e.g. in Phenix.Refine during the scaling of input intensities and subsequent outlier rejection according to expected intensity statistics). These processes took place completely automated as is the default in these programs and without any customization or user input.
Replication	All observations were made in at least two, typically three, independent experiments, which for numerical assays were each typically carried out with technical triplicates, all with consistent results.
Randomization	Randomization was not applicable as no experiments involving humans/animals were performed. Moreover, randomization was not applied as no experiment was sensitive to the order of measurement/treatment.
Blinding	Blinding was not carried out as no subjective analysis (e.g. scoring) was performed.

Reporting for specific materials, systems and methods

We require information from authors about some types of materials, experimental systems and methods used in many studies. Here, indicate whether each material, system or method listed is relevant to your study. If you are not sure if a list item applies to your research, read the appropriate section before selecting a response.

Materials & experimental systems

n/a	Involved in the study	
☐	☒	Antibodies
☐	☒	Eukaryotic cell lines
☒	☐	Palaeontology and archaeology
☒	☐	Animals and other organisms
☒	☐	Clinical data
☒	☐	Dual use research of concern

Methods

n/a	Involved in the study	
☒	☐	ChIP-seq
☒	☐	Flow cytometry
☒	☐	MRI-based neuroimaging

Antibodies

Antibodies used	Rabbit polyclonal anti-USP53 (Sigma-Aldrich, Cat# HPA035844; RRID: AB_10669532);<br>Mouse monoclonal anti-Tubulin, clone DM1A (Sigma-Aldrich, Cat# T6199; RRID: AB_477583);<br>Rabbit monoclonal anti-MARVELD2, clone 54H19L38 (Thermo Fisher, Cat# 700191; RRID: AB_2532298);<br>Mouse monoclonal anti-GAPDH, clone 6C5 (Thermo Fisher, Cat# AM4300; RRID: AB_2536381);<br>Sheep anti-mouse IgG, HRP-conjugated (Cytiva, Cat# NXA931; RRID: AB_772209);<br>Donkey anti-rabbit IgG, HRP-conjugated (Sigma-Aldrich, Cat# GENA934; RRID: AB_2722659);
Validation	Antibodies are validated for the application of Western Blotting on human proteomes per statements on the manufacturers' websites as referenced below.<br><br>The antibody for USP53 was validated by shRNA interference (Fig. 3c and Extended Data Fig. 4j). Moreover, the antibody was validated by siRNA (M-027186-01-0005, dharmacon) in multiple cell lines.<br><br>anti-Tubulin: https://www.sigmaaldrich.com/DE/de/product/sigma/t6199<br>anti-MARVELD2: https://www.thermofisher.com/antibody/product/MARVELD2-Antibody-clone-54H19L38-Recombinant-Monoclonal/700191<br>anti-GAPDH: https://www.thermofisher.com/antibody/product/GAPDH-Antibody-clone-6C5-Monoclonal/AM4300

Eukaryotic cell lines

Policy information about cell lines and Sex and Gender in Research

Cell line source(s)	HeLa (DSMZ, Cat# ACC 57; RRID: CVCL_0030);<br>Lenti-X 293T (TaKaRa, Cat# 632180; RRID: CVCL_4401);<br>CaCo-2 (ATCC, Cat# HTB-37; RRID: CVCL_0025);
Authentication	Cells were used without authentication.
Mycoplasma contamination	All cells were tested for mycoplasma contamination with a negative result.
Commonly misidentified lines<br>(See ICLAC register)	No commonly misidentified lines were used in this study.
