# Supplementary figures and images for: Discovery and mechanism of K63-linkage-directed deubiquitinase activity in USP53
Source: Nat Chem Biol. 2024 Nov 25;21(5):746–57. doi: 10.1038/s41589-024-01777-0 (PMC12037411; doi:10.1038/s41589-024-01777-0)

Uncropped gels and blots (Figure 1)

Fig. 1d

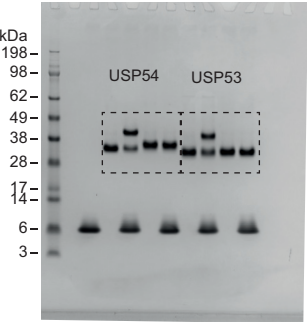

Fig. 1f

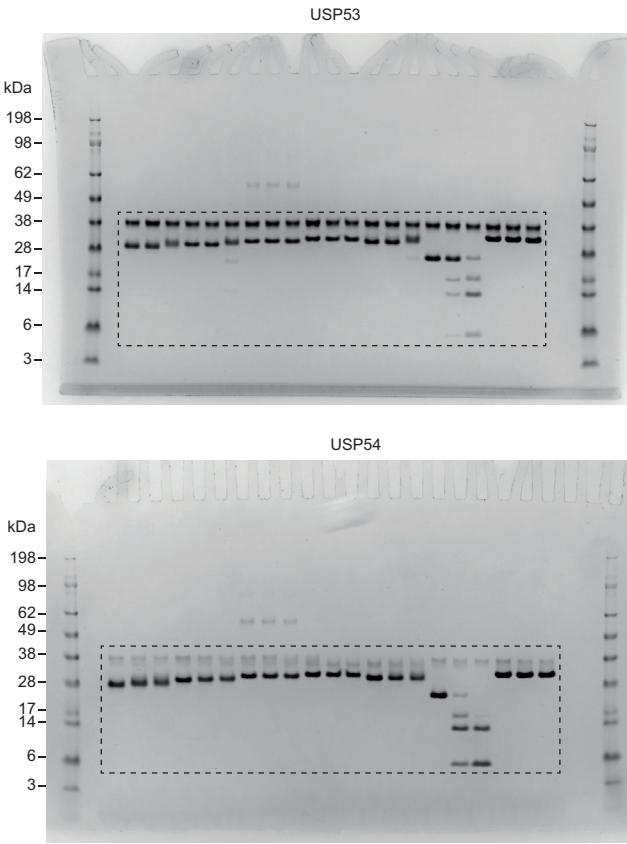

Supplement: Supplementary file 4 — Uncropped gels and blots. [file 41589_2024_1777_MOESM4_ESM.pdf]

Uncropped gels and blots (Figure 2)

Fig. 2b

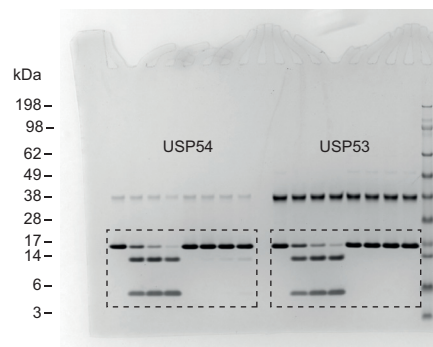

Fig. 2d

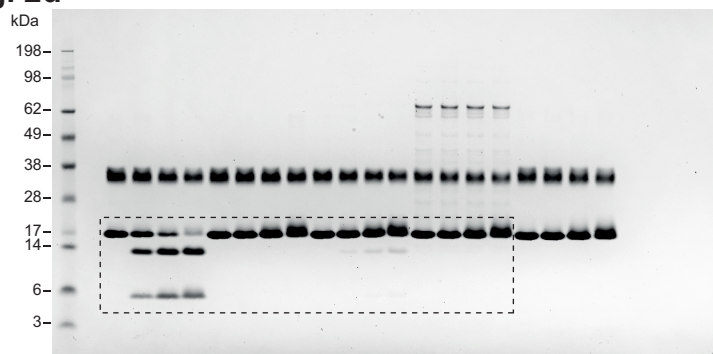

Fig. 2e

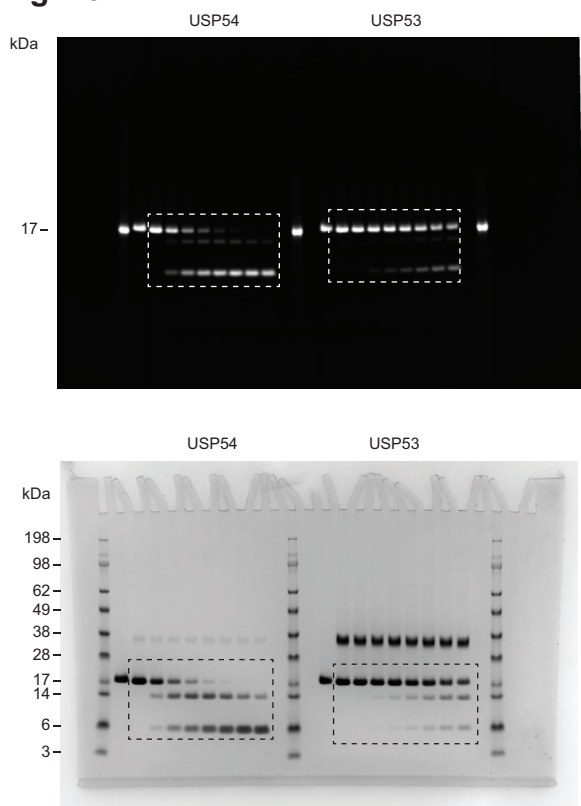

Supplement: Supplementary file 6 — Uncropped gels and blots. [file 41589_2024_1777_MOESM6_ESM.pdf]

## Uncropped gels and blots (Figure 4)

**Fig. 4d**

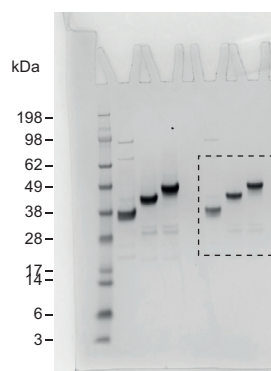

Supplement: Supplementary file 10 — Uncropped gels and blots. [file 41589_2024_1777_MOESM10_ESM.pdf]

## Uncropped gels and blots (Figure 5)

**Fig. 5g**

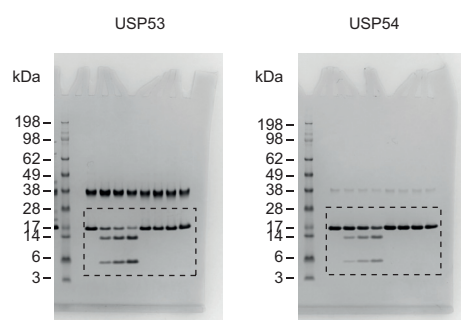

Supplement: Supplementary file 11 — Uncropped gels and blots. [file 41589_2024_1777_MOESM11_ESM.pdf]

## Uncropped gels and blots (Extended Data Figure 1)

Extended Data Fig. 1c

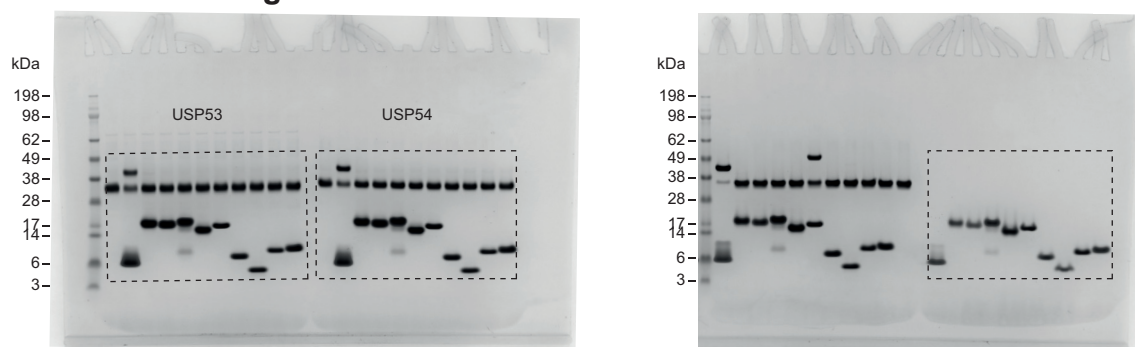

Supplement: Supplementary file 15 — Uncropped gels and blots. [file 41589_2024_1777_MOESM15_ESM.pdf]

## Uncropped gels and blots (Extended Data Figure 2)

Extended Data Fig. 2e

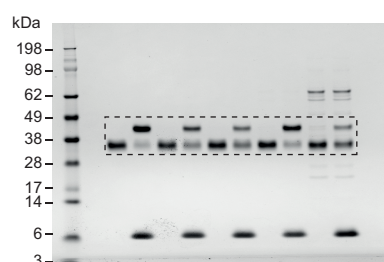

Extended Data Fig. 2f

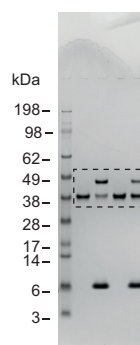

Supplement: Supplementary file 17 — Uncropped gels and blots. [file 41589_2024_1777_MOESM17_ESM.pdf]

## Uncropped gels and blots (Extended Data Figure 9)

Extended Data Fig. 9a

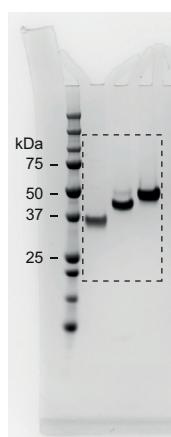

Supplement: Supplementary file 25 — Uncropped gels and blots. [file 41589_2024_1777_MOESM25_ESM.pdf]
