# Supplementary material for: Discovery and mechanism of K63-linkage-directed deubiquitinase activity in USP53
Source: Nat Chem Biol. 2024 Nov 25;21(5):746–57. doi: 10.1038/s41589-024-01777-0 (PMC12037411; doi:10.1038/s41589-024-01777-0)

Uncropped gels and blots (Figure 3, page 1)

Fig. 3a

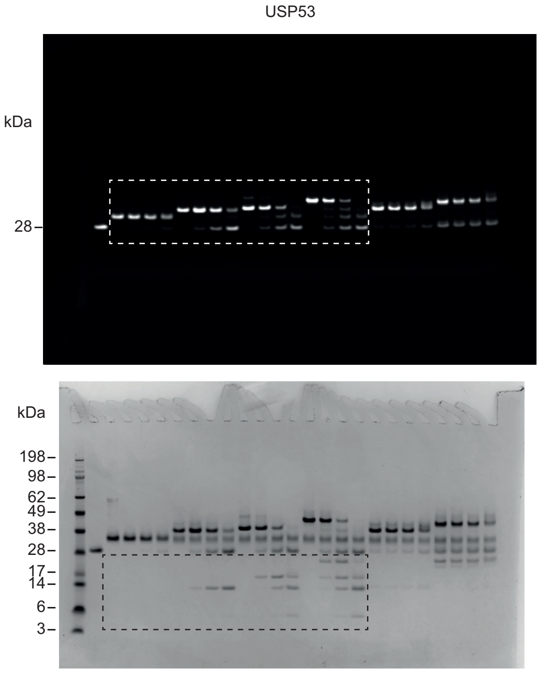

Fig. 3b

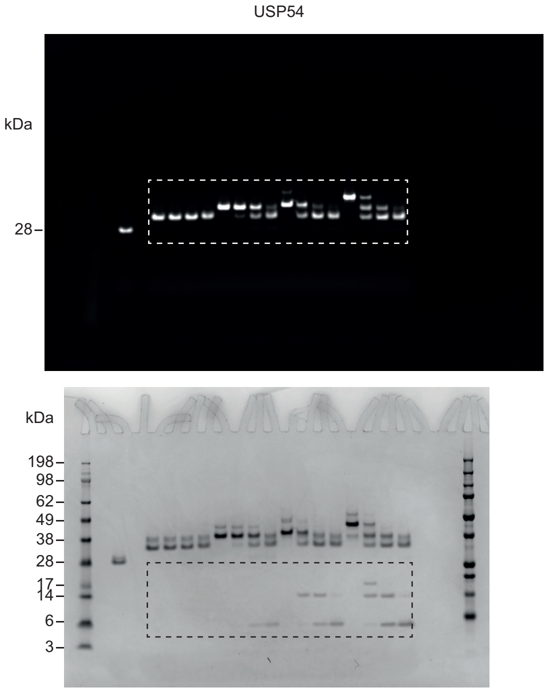

Fig. 3c

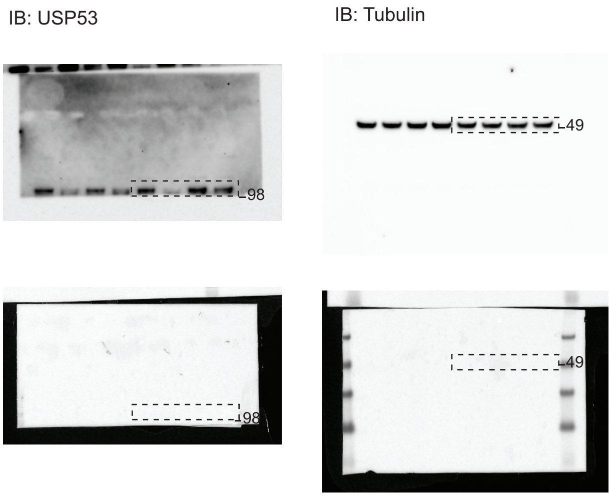

Uncropped gels and blots (Figure 3, page 2)

Fig. 3g

IB: MARVELD2

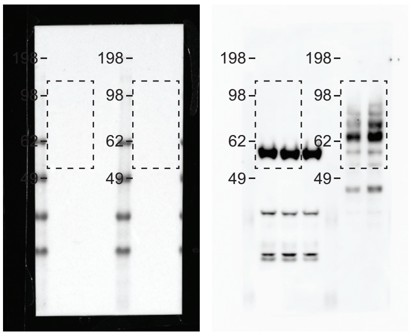

Fig. 3h

IB: MARVELD2

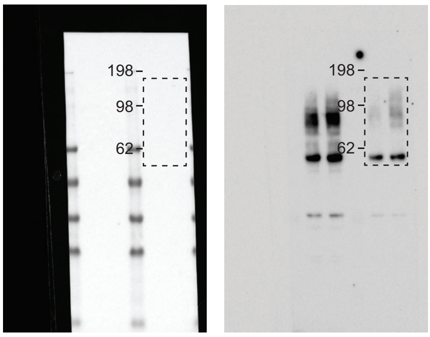

Fig. 3i

IB: USP53

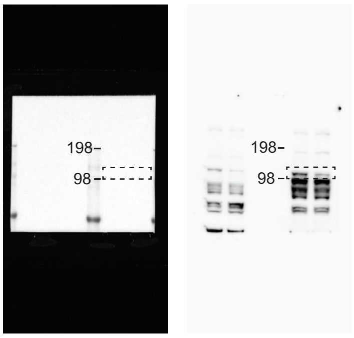

IB: GAPDH

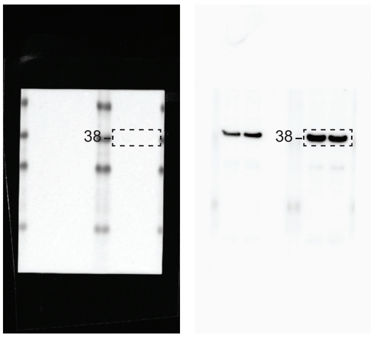

IB: MARVELD2

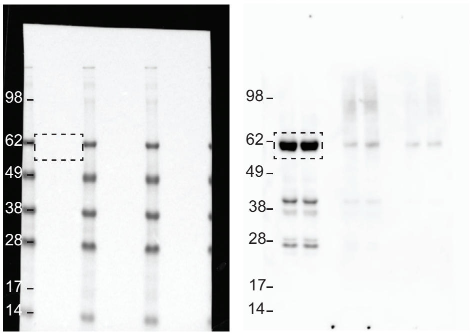

Fig. 3k

IB: MARVELD2

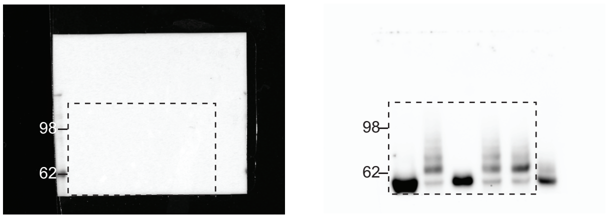

Fig. 3l

IB: MARVELD2

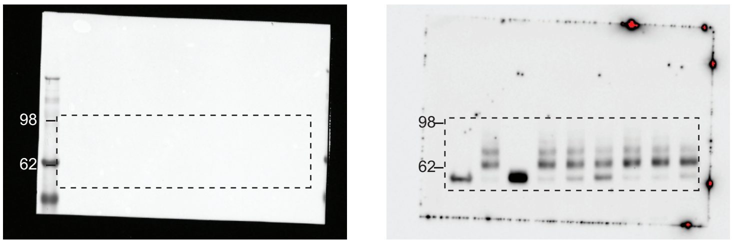

Supplement: Supplementary file 8 — Uncropped gels and blots. [file 41589_2024_1777_MOESM8_ESM.pdf]
