# Supplementary material for: Discovery and mechanism of K63-linkage-directed deubiquitinase activity in USP53
Source: Nat Chem Biol. 2024 Nov 25;21(5):746–57. doi: 10.1038/s41589-024-01777-0 (PMC12037411; doi:10.1038/s41589-024-01777-0)

Uncropped gels and blots (Figure 6, page 1)

Fig. 6d

Replicate I as shown in Fig. 6d

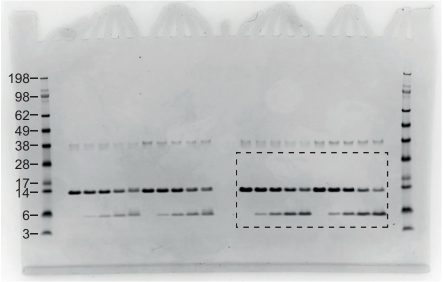

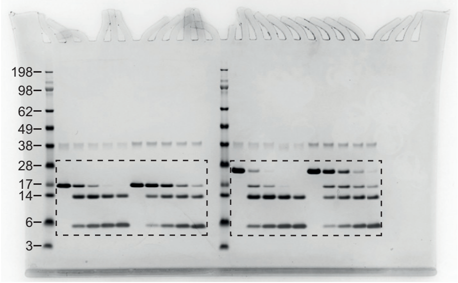

Replicate II

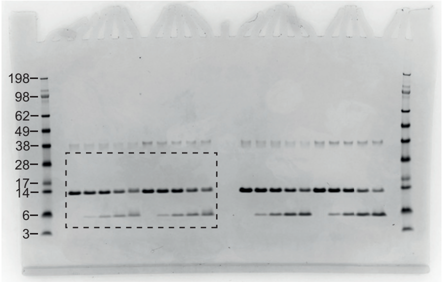

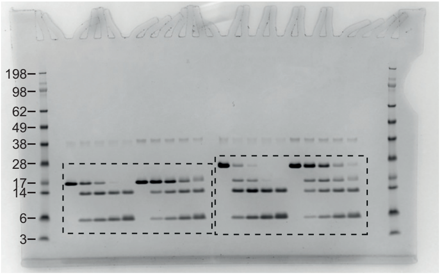

Replicate III

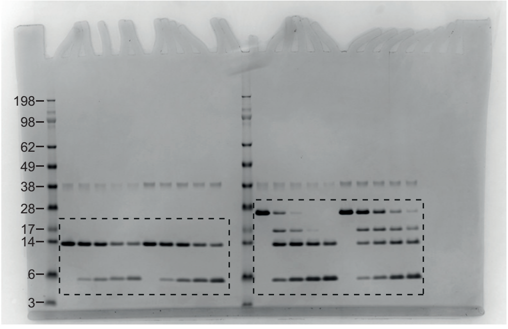

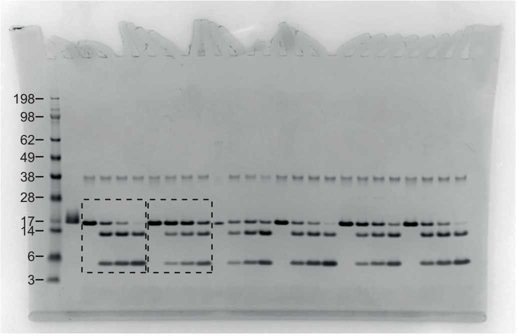

Uncropped gels and blots (Figure 6, page 2)

Fig. 6e

Replicate I as shown in Fig. 6e

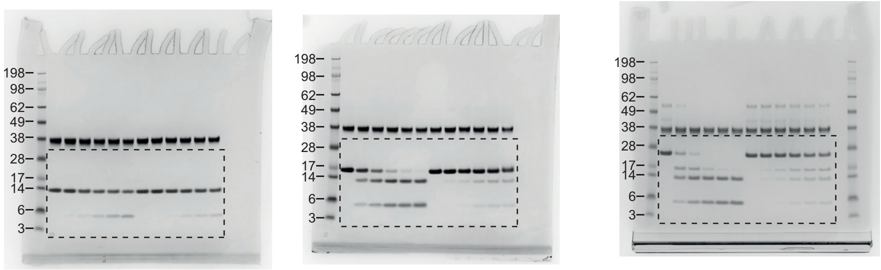

Replicate II

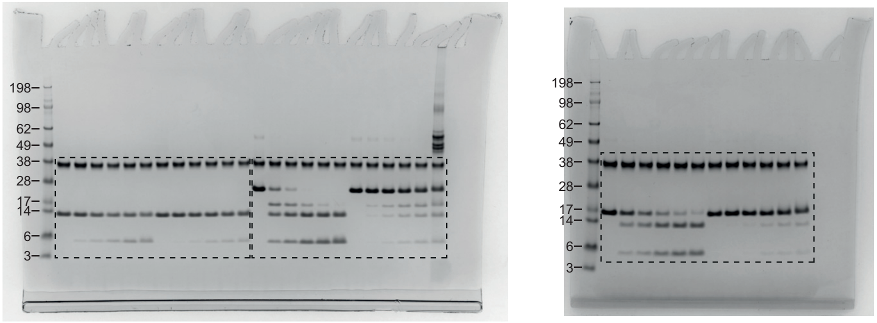

Replicate III

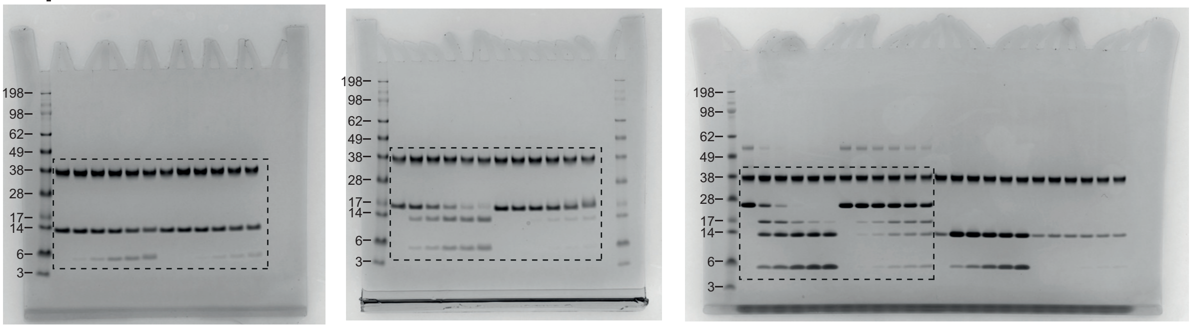

Supplement: Supplementary file 13 — Uncropped gels and blots. [file 41589_2024_1777_MOESM13_ESM.pdf]
