# Supplementary material for: Discovery and mechanism of K63-linkage-directed deubiquitinase activity in USP53
Source: Nat Chem Biol. 2024 Nov 25;21(5):746–57. doi: 10.1038/s41589-024-01777-0 (PMC12037411; doi:10.1038/s41589-024-01777-0)

Uncropped gels and blots (Extended Data Figure 3)

Extended Data Fig. 3a

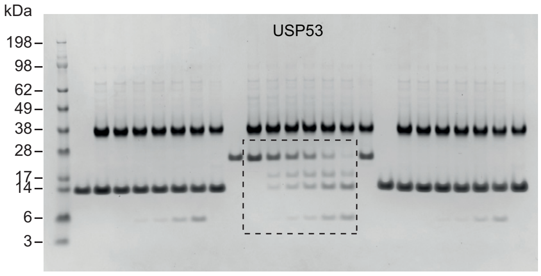

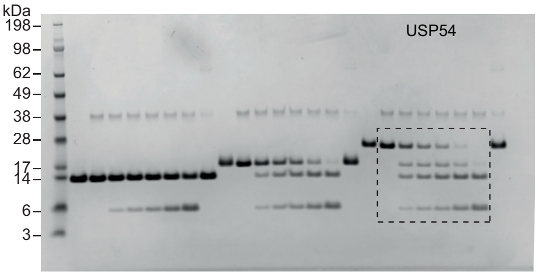

Extended Data Fig. 3c

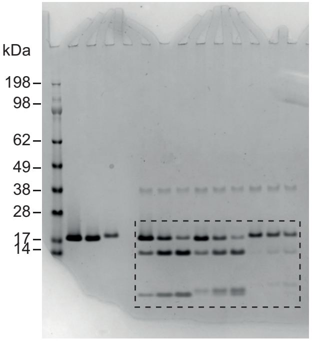

Extended Data Fig. 3e

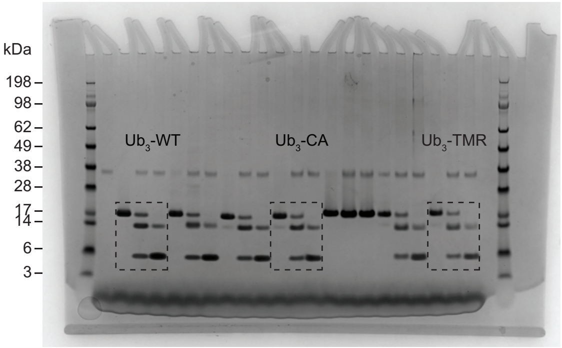

Extended Data Fig. 3g

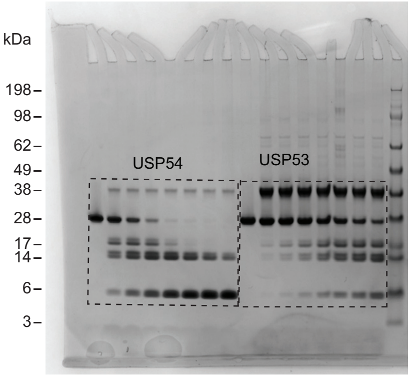

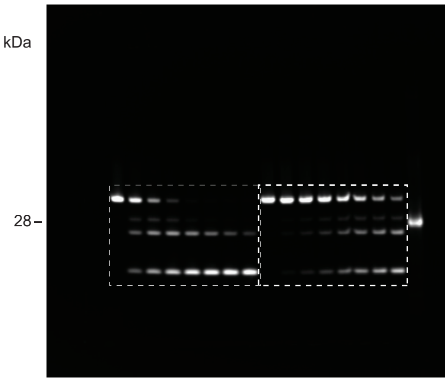

Supplement: Supplementary file 19 — Uncropped gels and blots. [file 41589_2024_1777_MOESM19_ESM.pdf]
