# Supplementary material for: Discovery and mechanism of K63-linkage-directed deubiquitinase activity in USP53
Source: Nat Chem Biol. 2024 Nov 25;21(5):746–57. doi: 10.1038/s41589-024-01777-0 (PMC12037411; doi:10.1038/s41589-024-01777-0)

Uncropped gels and blots (Extended Data Figure 4, page 1)

Extended Data Fig. 4c

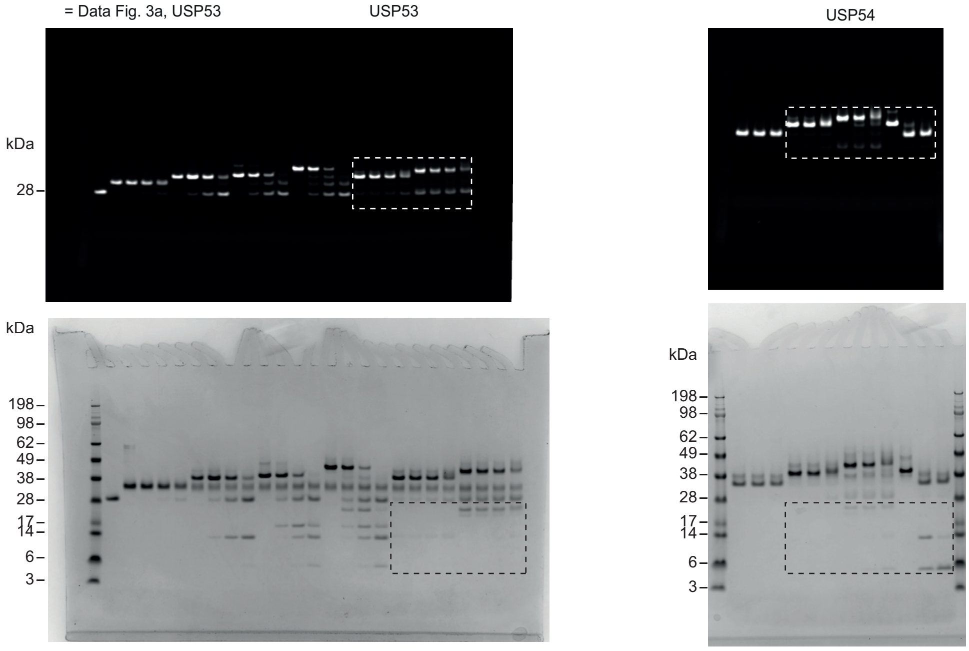

Extended Data Fig. 4g

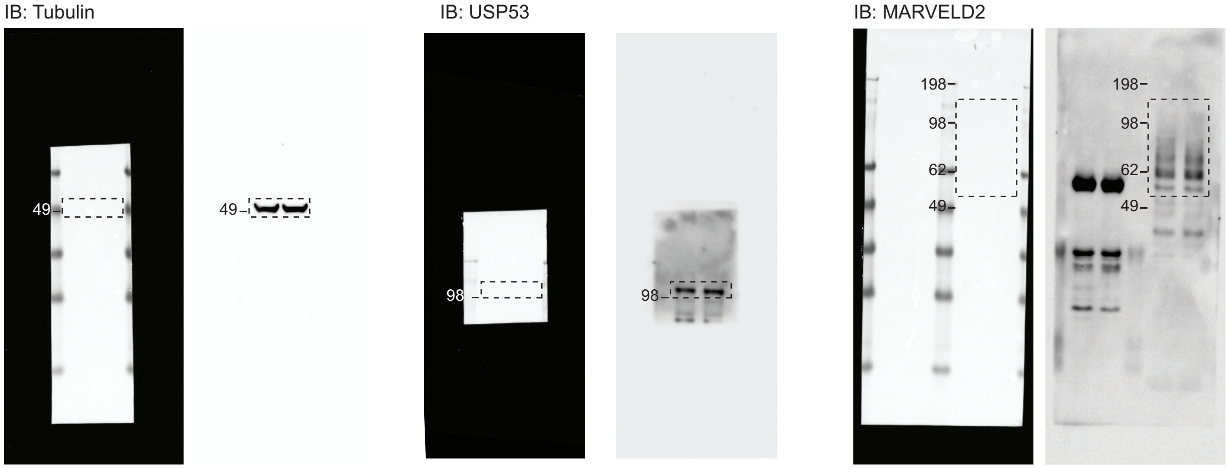

Extended Data Fig. 4h

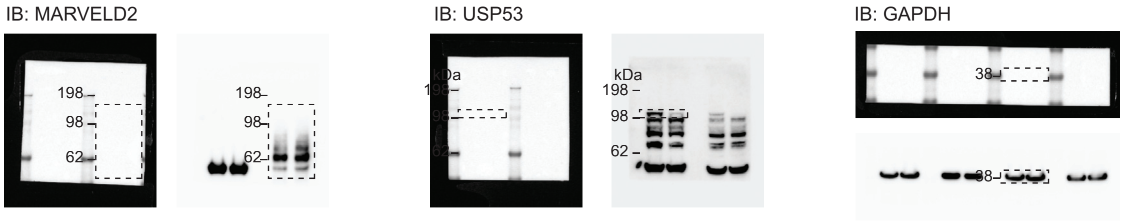

Uncropped gels and blots (Extended Data Figure 4, page 2)

Extended Data Fig. 4i

IB: GAPDH

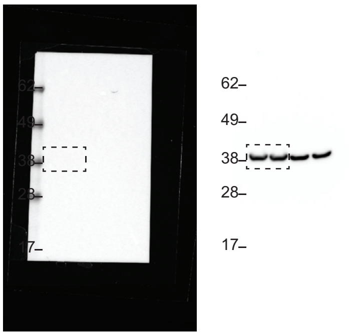

IB: USP53

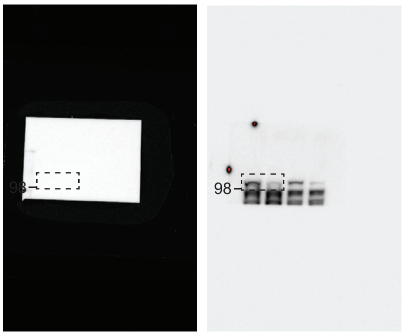

Extended Data Fig. 4j

IB: MARVELD2

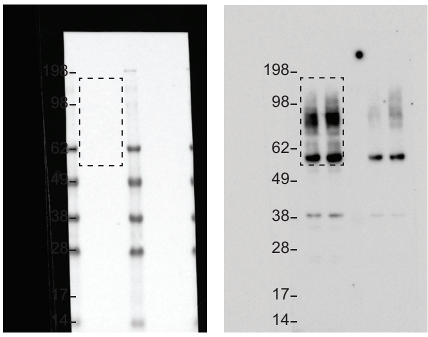

Extended Data Fig. 4k

IB: MARVELD2

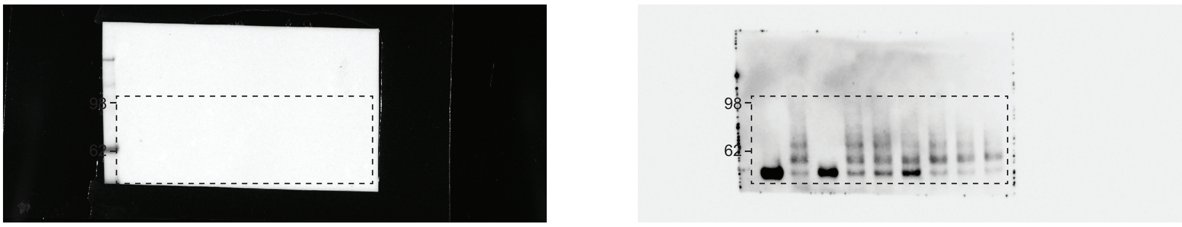

Supplement: Supplementary file 21 — Uncropped gels and blots. [file 41589_2024_1777_MOESM21_ESM.pdf]
